# Optimal path selection and secured data transmission in underwater acoustic sensor networks: LSTM-based energy prediction

**DOI:** 10.1371/journal.pone.0289306

**Published:** 2023-09-05

**Authors:** Sathish Kaveripakam, Ravikumar Chinthaginjala

**Affiliations:** School of Electronics Engineering, Vellore Institute of Technology, Vellore, India; Jaramogi Oginga Odinga University of Science and Technology, KENYA

## Abstract

The Underwater Acoustic Sensor Network (UASN) is a large network in which the vicinity of a transmitting node is made up of numerous operational sensor nodes. The communication process may be substantially disrupted due to the underwater acoustic channel’s time-varying and space-varying features. As a result, the underwater acoustic communication system faces the problems of reducing interference and enhancing communication effectiveness and quality through adaptive modulation. To overcome this issue, this paper intends to propose a model for optimal path selection and secured data transmission in UASN via Long Short-Term Memory (LSTM) based energy prediction. The proposed model of transmitting the secured data in UASN through the optimal path involves two major phases. Initially, the nodes are selected under the consideration of constraints like energy, distance and link quality in terms of throughput. Moreover, the energy is predicted with the aid of LSTM and the optimal path is selected with the proposed hybrid optimization algorithm termed as Pelican Updated Chimp Optimization Algorithm (PUCOA), which is the combination of two algorithms including the Pelican Optimization Algorithm (POA) and Chimp Optimization Algorithm (COA). Further, the data is transmitted via the optimal path securely by encrypting the data with the proposed improved blowfish algorithm (IBFA). At last, the developed LSTM+PUCOA model is validated with standard benchmark models and it proves that the performance of the proposed LSTM+PUCOA model attains 90.85% of accuracy, 92.78% of precision, 91.78% of specificity, 89.79% of sensitivity, 7.21% of FPR, 89.76% of F1 score, 89.77% of MCC, 10.20% of FNR, 92.45% of NPV, and 10.22% of FDR for Learning percentage 70.

## 1. Introduction

In recent years, Sensor Networks have surfaced as a viable research area. The routing issue is a vital component in these sorts of networks that must be addressed with the aim of prolonging the longevity of the company [1]. The amount of sensor nodes in the organization causes routing to become more and more unpredictable as the organization develops in size. Sensor networks used for underwater communication differ significantly from terrestrial sensor networks and even those used previously [2]. First, various programs need varying amounts of data, and second, networks operate on a shared platform rather than neglecting individual consumers. Instead of fairness among nodes, the goal is to optimize the performance. Third, there is a critical relationship between link distance, dependability, and hop count [3, 4]. Typically, packets over several short hops are preferred when compared to lengthy hops owing to energy optimization. This is because earlier research has shown that multi-hop data deliveries are ideal in terms of power consumption for underwater systems [5]. One of the most popular techniques for developing future sensor approaches and sea perception networks is wireless data transfer in ocean waters [6–8].

A few underwater acoustic communications are used in a variety of applications, including weather monitoring, atmospheric condition monitoring, and determining the amount of emissions, as well as shipping and ocean-related research [9]. Underwater sound communication is one of the most inventive methods of delivering and receiving underwater messages. This kind of transmission is useful for environmental monitoring, rapid response to natural disasters, pollution prevention, application of protection applications, the safety of off-shore ports and sectors and conducting various types of ocean and topographical research. Because of its numerous benefits, UASNs perform a vital and essential part in maritime innovation [10, 11].

Secure transmission is critical in UASN applications for information exchange and cooperation among sensor nodes. Acoustic channels are an open medium; hence, packets transmitted via UASNs can be easily intercepted [12]. Furthermore, the mobility of sensor nodes exposes the door to a variety of network security assaults. Because of the mobility of the nodes, communication between nodes in UASNs is extremely difficult. To avoid packet loss and malicious activity from affecting a node, it is critical to create a unique secure transmission mechanism [13–15]. Malicious nodes that do not forward a packet can also degrade network performance. Because of packet loss, routing overhead, and communication weaknesses, certain related techniques are unable to address or are inappropriate for strengthening encrypted communication in UASNs. Malicious nodes are continuously checked and are not authorized to take part in communication to improve transmission security [16]. In this regard, acoustic communication has been extensively embraced as a standard for underwater interaction by academics and industry. However, this technique has its drawbacks. Three significant issues exist. Initially, bandwidth is restricted and is affected by distance. Second, it is affected by time-varying multipath fading. Finally, sound travels at a slower rate than electromagnetic radiation [17–20].

In such a case, the usage of redundancy strategies such as Automatic Repeat Request (ARQ) protocols and Forward Error Correction (FEC) is required to provide error-free communication. However, using FEC alone is insufficient because an interface with a high bit error rate will end up resulting in several retransmissions. Furthermore, given the high Bit Error Rate (BER) and lengthy latency duration of underwater channels, it is difficult to achieve effective throughput for ARQ-based protocols [21–23]. Also, ARQ-based protocols have increased energy consumption and latency. ARQ methods have often been employed for high dependability in data delivery. The Stop and Wait (S&W) ARQ protocol, in particular, has been used as the only mechanism of retransmission in underwater acoustic sensor networks. It is related to the underwater acoustic modems’ half-duplexing mode [24]. However, the acoustic channel’s high BER and propagation latency make obtaining good throughput efficiency with the standard S&W ARQ challenging [25–27]. However, there are certain disadvantages associated with employing an underwater acoustic medium, such as a long end-to-end latency, a small bandwidth, and an elevated bit error rate [28]. Hence, this paper proposes a novel optimal path selection and secured data transmission in underwater acoustic sensor networks with LSTM-based energy prediction.

### 1.1 Problem statement

The features and challenges of extant works related to optimal path selection and secured data transmission in UASNs with different methodologies have shown in the above Table 1. Using the TSDBG method [1], the author achieved higher transmission latency but still it is necessary to describe the players’ trust value by comparing the behaviour of other nodes. With the Depth-Based Routing (DBR) and Grey Wolf Optimization (GWO) algorithm [2], the author achieved less delay and reduces the total count of dead nodes but concerning the mobile devices into the UWASNs is still in vague. In paper [3], the author provided an energy efficient reliable data transmission scheme for complex environmental observing in underwater acoustic sensor networks and it accomplished reduction in the communication overhead and energy consumption. However, still it is a challenge in predicting the leak point of marine oil pipelines. Though the author attained low complexity system with high efficiency using the DL-HDBT [4] based mechanism, still concerning the packet loss ratio is a challenging issue. Using paper [5], the author improved the efficiency of energy but designing a reliable and topology independent protocols are still a difficult problem. In paper [6], the author presented a novel energy efficient approach with quality of service aware based on routing with utilizing underwater wireless sensor networks and it utilized least amount of energy with enhanced network dependability. Yet, it is difficult in concerning the average packet latency and the network longevity. Using CARQ protocols [7], the author achieved the relay location but still it is a challenge to control the large propagation delay. The Fuzzy clustering Algorithm [8] is proposed by the author in his suggested cluster head selection approach in UWASN and obtained decreases in the death rate of the nodes. Wang et al. [29] designed the Edge prediction-based adaptive data transmission algorithm (EP-ADTA) with an end-edge-cloud architecture, aiming to develop a comprehensive data transmission algorithm considering application requirements. Alqahtani GJ et al. [30] introduced the Mobility prediction optimal data forwarding (MPODF) protocol for UASNs, incorporating a realistic and physically inspired mobility model, achieving high packet delivery ratio, energy efficiency, and reduced end-to-end delay. Anitha et al. [31] proposed an intelligent selection method for modulation schemes in UWA communication systems using a hybrid learning model, achieving a high accuracy rate and improved Bit Error Rate (BER) performance. Sathish et al. [32] proposed energy-balanced reliable and effective clustering for underwater wireless sensor networks. Yet, designing a lot of initial clustering algorithms to lower redundant data is still a challenging issue.

**Table 1:** The features and challenges of extant works in UASNs with different methodologies.

Author [Citation]	Methodology	Features	Challenges
Muthukkumar, R., and Manimegalai, D., [1]	TSDBG	It has higher transmission latency.	Need to describe the players’ trust value by comparing the behaviour of other nodes.
Gola, K.K., and Gupta, B., [2]	DBR GWO algorithm	It achieves less delay It reduces the total count of dead nodes.	It doesn’t concern the mobile devices into the UWASNs.
K. Wang, et al [3]	EGRC	It reduces the communication overhead. It reduces the consumption of energy.	It is still a challenge in predicting the leak point of marine oil pipelines.
Hemavathy, N., and Indumathi, P., [4]	DL-HDBT	It attains low complexity system with high efficiency.	It doesn’t concern the packet loss ratio.
Goutham, V., et al. [5]	HARQ method	It improves the efficiency of energy	Need to design a reliable and topology independent protocols
P. Sathya, & P. Sengottuvelan [6]	CSOA-EQ	It utilizes least amount of energy. It enhances network dependability.	It doesn’t concern the average packet latency and the network longevity.
Jamshidi, A., [7]	CARQ protocols	No need to aware about the relay location.	It is necessary to control the large propagation delay.
Krishnaswamy, V., & Manvi, S.S. [8]	Fuzzy clustering Algorithm	It decreases the death rate of the nodes.	Need to design several initial clustering algorithms to lower the redundant data.
Wang et al.[29]	Edge prediction-based adaptive data transmission algorithm (EP-ADTA)	- End–edge–cloud architecture	Developing a comprehensive data transmission algorithm considering application requirements
Alqahtani GJ et al. [30]	Mobility prediction optimal data forwarding (MPODF) protocol for UASNs	Realistic and physically inspired mobility model	Achieving high packet delivery ratio, energy efficiency, and reduced end-to-end delay
Anitha et al. [31]	Intelligent selection method for modulation schemes in UWA communication systems	Hybrid learning model combining CNNs and ensemble single feedforward layers	Achieving high accuracy rate and improved Bit Error Rate (BER) performance in underwater communication systems

### 1.2 Motivation

The research gaps like poor packet loss ratio, average packet latency, network longevity, delay, security and redundant data have been diminished in the research work via the proposed LSTM+PUCOA model. Since, the proposed LSTM+PUCOA model selects the optimal path via various constraints such as energy, distance and link quality. In addition, the security of the LSTM+PUCOA model is also enhanced via the improved blowfish algorithm. The motivation for this research on the UASN stems from the challenges posed by Energy prediction for optimal path selection, Hybrid optimization algorithm for path selection and Secured data transmission.

- Energy prediction for optimal path selection: In order to select an optimal path for data transmission in UASN, it is crucial to consider various constraints such as energy, distance, and link quality. Predicting the energy consumption of nodes can help in determining the most energy-efficient path for data transmission. In this research, Long Short-Term Memory (LSTM) is used to predict the energy levels of nodes in UASN.
- Hybrid optimization algorithm for path selection: The proposed model utilizes a hybrid optimization algorithm called Pelican Updated Chimp Optimization Algorithm (PUCOA) to select the optimal path. PUCOA combines two algorithms, namely Pelican Optimization Algorithm (POA) and Chimp Optimization Algorithm (COA). This hybrid approach aims to improve the efficiency and effectiveness of path selection in UASN.
- Secured data transmission: Ensuring the security of data transmission is crucial in UASN to protect sensitive information from unauthorized access. The proposed model incorporates an improved blowfish algorithm to encrypt the data, thus enhancing the security of the transmitted data.

### 1.3 Contributions

The contributions of the proposed model for optimal path selection and secured data transmission in the UASN can be summarized as follows:

- Develop a hybrid optimization algorithm PUCOA, which selects the optimal path in UASN with the consideration of constraints such as energy, distance and link quality in terms of throughput.
- Develop an improved blowfish algorithm to encrypt the data for ensuring secured transmission.
- Utilization of LSTM for energy prediction, capturing temporal dependencies and patterns in energy consumption data to accurately predict future energy levels.
- Secure data transmission through the optimal path achieved by encrypting the data with the proposed improved Blowfish algorithm.
- Validation of the developed LSTM+PUCOA model using standard benchmark models, demonstrating high performance with metrics such as 93.78% accuracy, 95.38% precision, and 95.85% specificity.

The organization of this work is as follows: Section 2 explained the extant works of the underwater acoustic sensor networks. The proposed secured data transmission in UASNs via LSTM-based energy prediction is explained in section 3. Section 4 describes the proposed model of secured data transmission via an improved blowfish algorithm. The experimental analysis related to this proposed model is demonstrated in section 5 and the conclusion of this proposed work is summarized in section 6.

## 2. Literature review

In 2021, Muthukkumar, R., and Manimegalai, D., [1] implemented a secured transmission with trust based dynamic Bayesian game in UASN. In the network, a preserved data was generated over the nodes. To determine whether the specified node participated in the packet dropping or involved in misbehaving performance could be computed by evaluating payoff and trust for each node at the time of transmitting the data. With the aid of Bayes’ rule, the trust value for all nodes was modified. In accordance with the trust value, the usual nodes are observed simultaneously to identify the neighbour nodes. Further, the estimated outcome illustrated that the presented approach minimized the attack of packet dropping, propagation delay. Hence, the proposed model, improved their performance in secure transmission of data.

In 2021, Gola, K.K., and Gupta, B., [2] proposed a void area avoidance routing and energy efficient with the Grey Wolf optimization algorithm. The optimal forwarder node was selected with the proposed Grey wolf optimization algorithm. The suggested approach improved the lifetime of network with rejecting the void region and this balanced the network energy effectively. Moreover, the presented method accomplished better packet delivery ratio and also reduced the mean dead nodes amount with end-to-end delay. Thus, the proposed approach enhanced the entire network lifetime and lowered the transmission delay.

In 2016, K. Wang, et al. [3] provided an energy efficient reliable data transmission scheme for complex environmental observation in underwater acoustic sensor networks. It utilized energy efficiency Grid routing based on 3D cubes approach that handled complicated properties like high transmission delay, density and 3D changing topology. The sink node was the agent that directed the data packets to the destination side. The data was processed by the agent that eliminated the data redundancy and chooses the further point, which wanted to visit and thereby it employed locations of sensor node, residual energy and end-to-end delay.

In 2021, Hemavathy, N., and Indumathi, P., [4] introduced a deep learning based Hybrid Dynamic Biased Track (HDBT) routing protocol for underwater acoustic sensor networks. The proposed approach was the combination of both deep learning and hybrid dynamic biased track method. The optimal relay nodes were found by the proposed deep learning method in the network environment. Also, with the high dynamic bias track, the traffic congested nodes were tracked. In the ns2-AqaSim simulator, the routing protocol was established. Thereby, the presented method accomplished maximum throughput in nodes and performed well in balance positioning.

In 2021, Goutham, V., et al. [5] developed a hybrid ARQ method for incremental cooperative communication in UASN. At the time of transmitting the packet, the suggested method employed the neighbourhood sensor nodes. The presented approach merged the hybrid automatic repeat request scheme with the incremental cooperative communication. The size of the packet and the modulation level were act as function of distance and leveraged in between the source and destination, to increase the energy efficiency by the proposed optimization algorithm. Thus, the presented approach achieved better efficient in energy in correspond to the alteration in parameters such as shipping noises and waves.

In 2022, P. Sathya, & P. Sengottuvelan [6] presented a novel energy efficient approach with quality of service aware based on routing with utilizing underwater wireless sensor networks. The path was selected with the proposed approach by establishing differential equation in the suggested method in accordance with the residual energy and energy utilization. The crucial demonstration in the proposed system was the fuel efficiency. Further, the route was selected by the proposed cuckoo search optimization algorithm, in accordance with the residual energy and pheromone

In 2019, Jamshidi, A., [7] implemented a novel Co-operation based Automatic Repeat Request (CARQ) protocols to select the alternate path with the intermediate nodes in between the source and destination. With this presented method, the relay locations were considered by generating a time based schedule in the destination. Moreover, they computed the efficiency of throughput by comparing the results with the conventional ARQ method like stop and wait and also compared with the traditional methods in terms of throughput. Also, the presented method achieved better performance by attaining maximum throughput.

In 2019, Krishnaswamy, V., & Manvi, S.S. [8] suggested a cluster head selection approach with the hierarchical network control in UWASN. With the aid of a fuzzy clustering algorithm, the node clustering was performed in accordance with the geographical positions. Moreover, the cluster head selection process was performed with the aid of improved Particle Swarm Optimization (PSO), under the consideration of the distance between the nodes and less energy consumption. Also, the proposed method attained lower in death rate of the nodes. Thus, the proposed approach prolonging for lifespan of the topology.

In 2022, Wang et al. [29] designed the routing algorithm solely based on the transmission environment is insufficient. Instead, a comprehensive data transmission algorithm should be developed by considering the application requirements as well. To address these challenges, the paper proposes an edge prediction-based adaptive data transmission algorithm (EP-ADTA). EP-ADTA employs the end–edge–cloud architecture to define the underwater wireless sensor networks. The algorithm treats communication nodes as agents, enabling monitoring data prediction and compression based on edge prediction. It dynamically selects the transmission route and controls the data transmission accuracy using reinforcement learning. Simulation results demonstrate that EP-ADTA can meet the accuracy requirements of underwater monitoring applications, adapt to changes in the transmission environment, and ensure efficient and reliable data transmission in underwater wireless sensor networks.

In 2021, Alqahtani GJ et al. [30] Proposed a mobility prediction optimal data forwarding (MPODF) protocol for UASNs, leveraging mobility prediction techniques. The protocol incorporates a realistic and physically inspired mobility model to predict sensor node movements accurately. By using this mobility prediction module, the MPODF protocol successfully forwards every generated data packet through a single best path without the need for exchanging notification messages. Simulation results demonstrate the effectiveness of the MPODF protocol in achieving a high packet delivery ratio, energy efficiency, and reduced end-to-end delay. The protocol ensures that data packets are reliably delivered to their intended destinations, conserves energy resources, and minimizes the time required for data transmission. The proposed protocol addresses the challenges posed by underwater environments and provides an efficient solution for data forwarding in UASNs.

In 2023, Anitha et al., [31] proposed a novel intelligent selection method for modulation schemes in UWA communication systems. The modulation schemes considered in the study are Code Division Multiple Access (CDMA), Time Division Multiple Access (TDMA), and Orthogonal Frequency Division Multiplexing (OFDM). The proposed method combines the power of convolutional neural networks (CNNs) and ensemble single feedforward layers (SFL) to achieve intelligent modulation selection. The CNNs are employed to extract relevant features from the underwater acoustic channels. These features capture the unique characteristics of the channel, such as propagation delay and Doppler shifts. The extracted features are then used as inputs to the ensemble single feedforward layers, which make the modulation selection based on the outputs of the CNNs. Extensive experimentation and simulations are conducted to evaluate the performance of the proposed hybrid learning model. The results are compared with other hybrid learning models and conventional methods commonly used in underwater communication systems. The simulation results demonstrate that the proposed hybrid learning model achieves an impressive accuracy rate of nearly 98% and a 30% improvement in Bit Error Rate (BER) performance compared to other learning models. These results indicate that the proposed method outperforms alternative approaches and effectively achieves reliable communication schemes in dynamic underwater environments.

Based on the literature review as represented in Table 1, the research gaps like security and optimization have been diminished in the research work via the proposed LSTM+PUCOA model covers efficient path selection and secure data transmission in UASNs with LSTM-based energy prediction. UASNs face issues including limited energy and data transfer. They suggest using LSTM networks to forecast underwater sensor node energy consumption and identify energy-optimal data transmission methods. The LSTM-based energy prediction model allows proactive energy management and optimal path selection, extending the network’s lifetime. Encryption and authentication techniques maintain data security and integrity in UASNs. The literature review shows how LSTM-based energy prediction techniques can optimize path selection and secure data transmission in UASNs, improving their performance and reliability in challenging underwater environments.

## 3. Proposed secured data transmission in UASNs via LSTM-based energy prediction

### 3.1 System model

Assume a simple UASNs environment with three types of nodes as normal nodes, intermediate nodes and advanced nodes. Consider a normal nodes *N*_*i*_, intermediate nodes *I*_*i*_ and advanced nodes *A*_*i*_, where *i* = {1, 2, …. *n*}. The advanced nodes allotted in the network with the fixed coordinates. On the other hand, the normal nodes are randomly distributed in an environment. The advanced nodes provide lot of source energy when compared to the normal nodes. That is, the initial energy of advanced nodes is 1J and the initial energy of normal nodes is 0.5J. Thereby, the network lifetime has maximized essentially. Also, the intermediate nodes are performed in between the normal and advanced nodes and the initial energy of intermediate nodes is 0.8J. The sensor nodes are randomly distributed that makes the communication link reliable. There exist a sink, which has unlimited power and energy consumption. This carries out wireless transmission. The distance between the nodes is calculated according to the signal transmission strength.

**Energy prediction using LSTM**: The energy level of each node is predicted via deep learning algorithm based on specific features including location of the node *l*_*N*_, distance *Dist* and the node type *N*_*T*_: *N*_*T*_ ∈ {*N*_*i*_, *I*_*i*_, *A*_*i*_}. Accordingly, the three types of nodes like *N*, *I*_*i*_ and *A*_*i*_ set the energy value as follows:

$$\begin{array}{ccc} \text{Node}\;\text{type} & \text{Target} & \text{Energy}\;\left(\text{J}\right)\\ (\text{i})\;\text{Normal}\;\text{node}\;N_{i} & 0 & 0.5\\ (\text{ii})\;\text{Intermediate}\;\text{node}\;I_{i} & 1 & 0.8\\ (\text{iii})\;\text{Advanced}\;\text{node}\;A_{i} & 2 & 1 \end{array}$$

**Fig 1:**
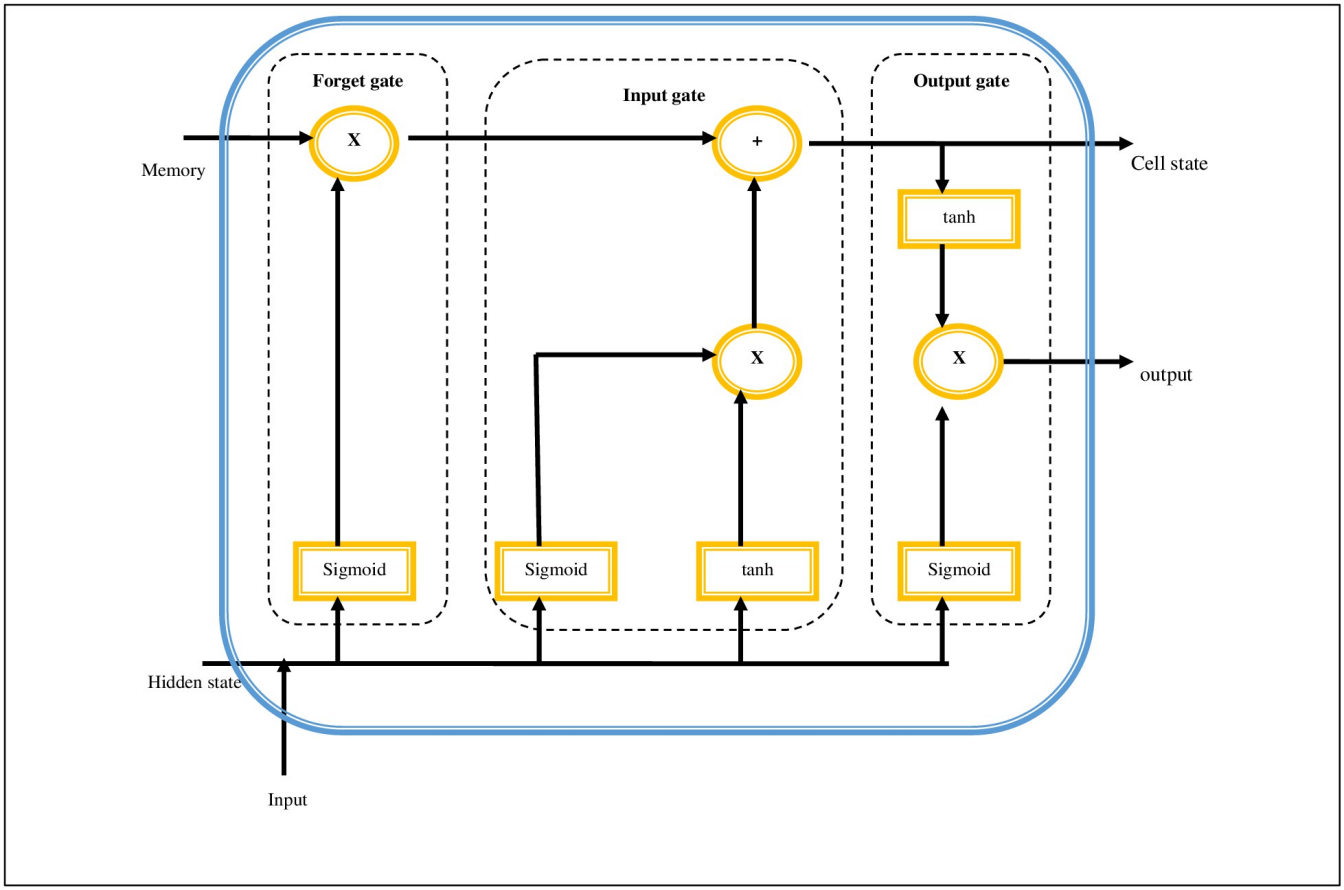
Architecture of LSTM.

The input to the LSTM [24] involves the following features such as *l*_*N*_, *Dist* and *N*_*T*_ to predict the energy. The feature *fs*: *fs* = [*l*_*N*_, *Dist*, *N*_*T*_] is fed into the LSTM neural network at the input gate. “Fig 1” depicts the architecture of the LSTM. The neural network LSTM includes three major gated units like input gate *Ig*_*t*_, forget gate *Fg*_*t*_ and output gate *Og*_*t*_. These gated units remember the data series and keep the information for a long time series and accomplishes greater accuracy in prediction. The input gate can be stated as in Eq (1). Here, *w*_*i*_ specifies weight if the input gate *h*_*t*−1_ specifies output state of the hidden layer, *b*_*i*_ specifies the bias vector of input gate and *S* specifies sigmoid function.


$$Ig_{t}=S\left(w_{i}*\left[h_{t-1}fs_{t}\right]+b_{i}\right)$$ (1)

The forget gate can be stated as in Eq (2). Here, *w*_*f*_ specifies weight of the forget gate and *b*_*f*_ specifies bias vector of forget gate.


$$Fg_{t}=S\left(w_{f}*\left[h_{t-1}fs_{t}\right]+b_{f}\right)$$ (2)

The output gate can be stated as in Eq (3). Here, *w*_*o*_ specifies weight of the output gate, and *b*_*o*_ specifies bias vector of output gate.


$$Og_{t}=S\left(w_{o}*\left[Cg_{t},h_{t-1},fs_{t}\right]+b_{o}\right)$$ (3)

The candidate gate is updated with the hyperbolic tangent can be stated as in Eq (4). Here, tanh specifies hyperbolic tangent function and *w*_*c*_ specifies weight of the candidate gate.


$$Cg_{t}=Fg_{t}*Cg_{t-1}+Ig_{t}*\text{tanh}\left(w_{c}*\left[h_{t-1},fs_{t}\right]\right)$$ (4)

Also, the hidden state is updated with the hyperbolic tangent function can be stated as in Eq (5).


$$h_{t}=Og_{t}*\text{tanh}\left(Cg_{t}\right)$$ (5)

### 3.2 Optimal path selection using proposed PUCOA

**Fig 2:**
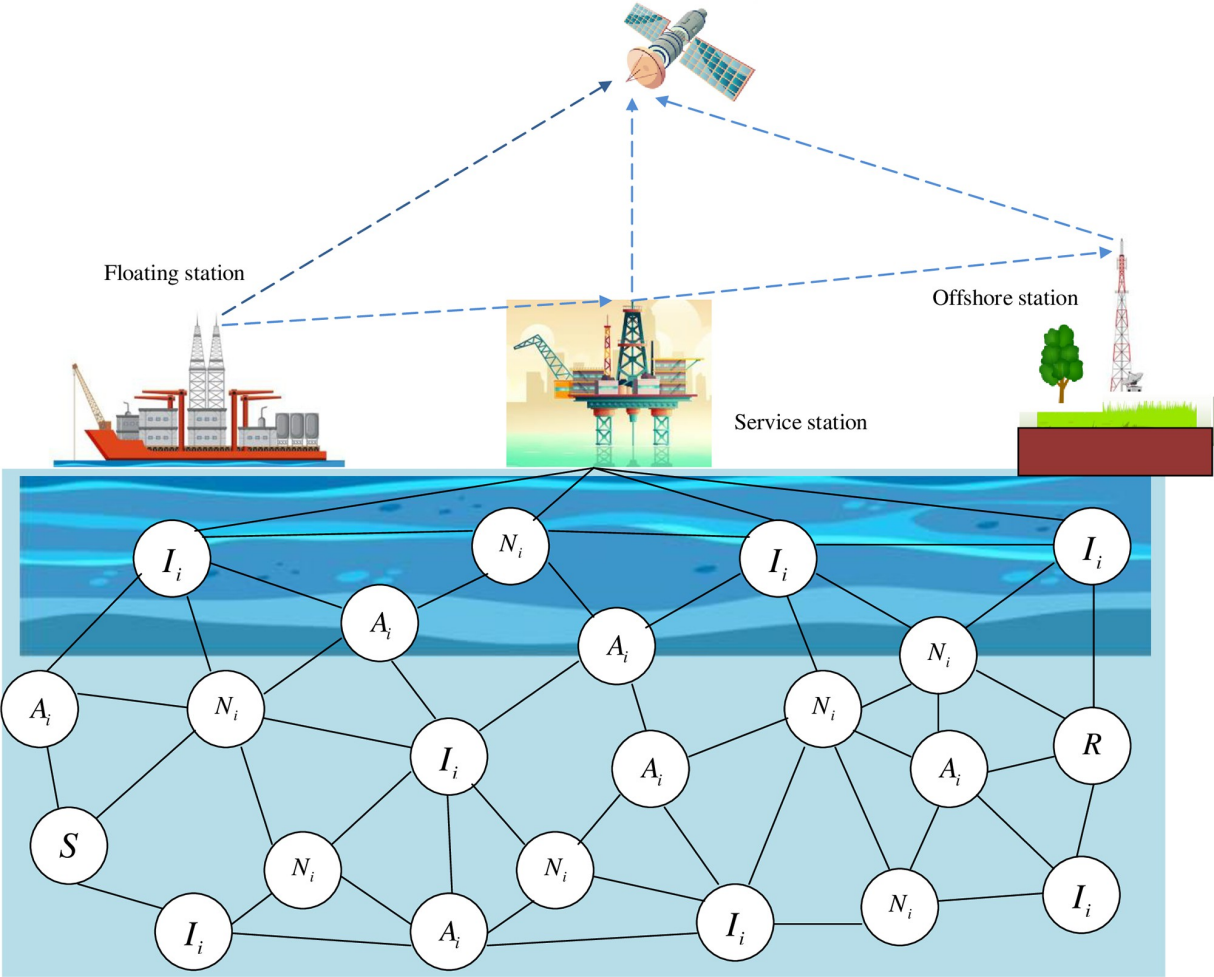
The three categories of node between the sender and receiver nodes in underwater acoustic sensor network.

**Fig 3:**
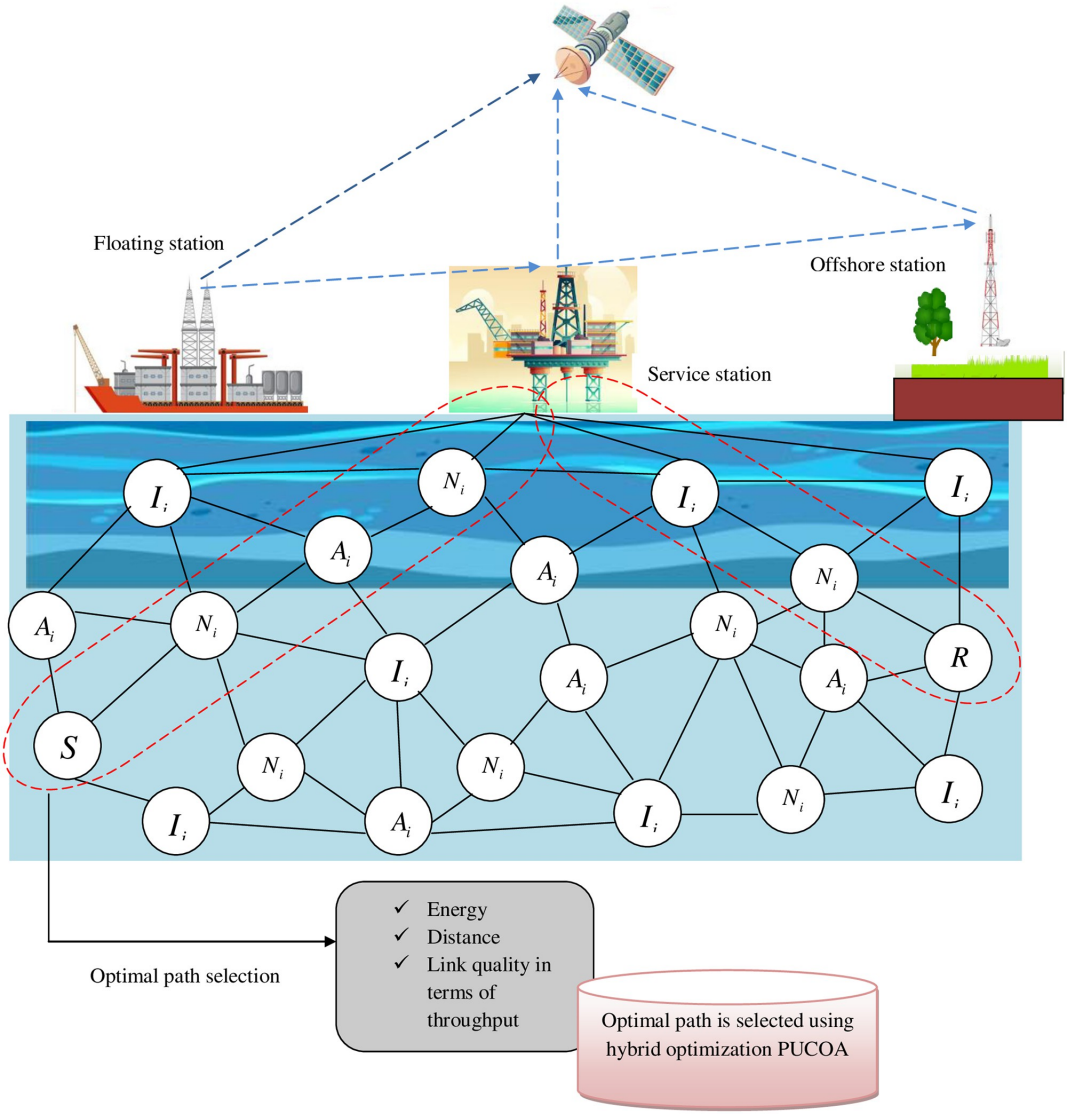
The process of optimal path selection by considering the shortest path distances with the proposed hybrid optimization algorithm PUCOA.

In UASNs, there are several nodes between the sender *S* and receiver *R* node. In this work, it classifies the node into three categories: they are normal node, intermediate node and advanced node that has been depicted in the following “Fig 2”. The nodes are selected with the consideration of constraints such as energy, distance, and link quality. Among those selected nodes, the optimal paths are needs to be selected with the proposed hybrid optimization algorithm PUCOA. The proposed hybrid optimization algorithm PUCOA is the combination of both pelican optimization algorithm and chimp optimization algorithm. The following “Fig 3” shows the representation of selected shortest path by the proposed hybrid optimization algorithm PUCOA. Once the optimal path is selected, then the data packets are transmitted via the optimal paths. To transmit the data securely, this paper employs an improved blow fish algorithm to maintain the secrecy of the data by following encryption.

### 3.3 Objective function

The objective function considered to choose the optimal path is given here as in Eq (6). Here, *E* indicates energy, *D* indicates the distance, *Lq* indicates link quality and (*W*_1_, *W*_2_ and *W*_3_) are the weights coefficients such that ∑*W*_*i*_ = 1.


$$Obj=\text{min}\left(W_{1}*\left(1-E\right)\right)+W_{2}*D+W_{3}*\left(1-Lq\right)$$ (6)

### 3.4 Energy

This work utilizes the energy consumption model [2] for underwater acoustic data transmission. In the sender side, for sending a data consumes more energy than the receiver side to receive the data. By reducing the energy consumption of sender side, the overall energy consumption for the network is also get reduced. Consider the minimum power that is essential to receive the *l* bit data and the energy consumes for sender side can be stated as in Eq (7). Here, *S*_*d*_ indicates the sending delay node, *A*(*x*) indicates the function assisted to underwater acoustic propagation model and *P*_0_ indicates power.


$$E\left(l,x\right)=S_{d}A\left(x\right)P_{0}$$ (7)

Then the function assisted to underwater acoustic propagation model can be stated as in Eq (8). Here, *a* indicates frequency related variable and *k* indicates parameter assisted to the underwater acoustic propagation model.


$$A\left(x\right)=a^{k}x^{k}$$ (8)

Thus the energy consumption for each node can be estimated as in Eq (9). Here, *nE* indicates node energy.


$$E(l,x)=Mean\left(nE\right)$$ (9)

### 3.5 Distance

The distance [16] is computed between the nodes with their coordinates (*x*_1_, *y*_1_) and (*x*_2_, *y*_2_), respectively. In this work, consider 100 nodes are available in underwater acoustic sensor network and the nodes are selected with the consideration of energy a link quality. The node distance is computed between the selected node (i.e. assume one node is at location 4 and another node is at the location 25). Among these nodes, the distance is computed that can be utilized for data packet delivering in a shortest path. Then the distance formulation can be stated as in Eq (10).


$$Dist=mean\left(dis\right)$$ (10)

Where, *dis* is the distance between the nodes that can be computed as in Eq (11).


$$dis=\sqrt{{\left(x_{2}-x_{1}\right)}^{2}+{\left(y_{2}-y_{1}\right)}^{2}}$$ (11)

### 3.6 Link quality in terms throughput

The link quality ensures the quality of the data packets received at the receiver side. In this paper, the link quality is computed in terms of throughput. The measure of overall units of the system details, which are processed at the given time, is termed as throughput. The formulation of throughput can be stated as in Eq (12). Here, *P*^*R*^ specifies packets received at each node and *P* specifies total number of packets.


$$Thp^{*}=\frac{sum\left(P^{R}\right)}{2P}$$ (12)

### 3.7 Proposed PUCOA hybrid optimization algorithm

The proposed PUCOA hybrid optimization is employs in this work to select the optimal path. Consider, the underwater acoustic network environment with 100 nodes, certain nodes is selected randomly for optimal path selection, among the 100 nodes under the consideration of constraints. In between the sender and receiver nodes (i.e. 1 and 100), the nodes are selected, among them the shortest path is need to be selected to transmit the data packets by using PUCOA hybrid optimization algorithm. The hybrid optimization utilizes the COA [23] strategy of implementing the search space independently. This optimization inspires by the behaviour of chimp that has dissimilar activities in terms of smartness and capability. The dissimilar activities of each chimp have been utilized in a specified condition. Moreover, The COA is updated with the Pelican optimization algorithm by inspiring the behaviour of pelicans. The strategy of pelicans has the ability to search the optimal solution in accordance with the constraints. However, this provides the quasi-optimal solution. To obtain the global optimal solution, a novel PUCOA hybrid optimization is proposed.

#### 3.7.1 Mathematical modelling of proposed hybrid optimization PUCOA

The mathematical modelling of proposed PUCOA describes the four kinds of chimps in a chimp settlement. They are driver, chaser, attackers and barriers. The ability of drivers chases the prey but fail to catch up it. The chasers have the capability of following the prey rapidly and readily catch up it. The attackers foretell the preys’ path to the chasers and inflict the prey and eventually, a dam is implemented by the barriers, over the prey progression. It includes four types of phases: they are exploitation, utilization, exploitation and sexual motivation phase. In the exploitation phase, the attacking approach is employed. The prey attacking approach is deploys in utilization phase. The prey searching and social incentive takes place in exploration and sexual motivation phase, respectively.

*3*.*7*.*1*.*1 Driving and chasing the prey*. The driver chase the prey without attempt to catch up it; whereas, the chaser follow the prey rapidly and catch up the prey. The mathematical formulation of driving and chasing the prey can be stated as in Eq (13). Here, *x*_*pr*_ specifies position of prey, *x*_*ch*_ specifies position of chimp and *t* specifies the current iteration set.


$$D=\left|b.x_{pr}\left(t\right)-g.x_{ch}\left(t\right)\right|$$ (13)

The position of chimp with respect to driving and chasing the prey can be stated as in Eq (14). Here, *c*, *g* and *b* specifies the coefficient vectors and their values are *c* = 2. *f*. *r*_1_ − *f*, *b* = 2.*r*_2_ and *g* = *chaotic*_*value*.


$$x_{ch}\left(t+1\right)=x_{pr}\left(t\right)-c.D$$ (14)

Further computing the above Eq (14), we get the modified position that can be stated as in below Eq (15).


$$x_{ch}\left(t+1\right)=x_{pr}\left(t\right)-c.\left|b.x_{pr}\left(t\right)-g.x_{ch}\left(t\right)\right|$$ (15)

By evaluating |*b*.*x*_*pr*_(*t*) − *g*.*x*_*ch*_(*t*)|, the following derivations follow:

$$\begin{array}{ll} \left|b.x_{pr}\left(t\right)-g.x_{ch}\left(t\right)\right| & =\sqrt{{\left(b.x_{pr}\left(t\right)\right)}^{2}+{\left(g.x_{ch}\left(t\right)\right)}^{2}-\left[2.\left(b.x_{pr}\left(t\right)\right).\left(g.x_{ch}\left(t\right)\right)\text{cos}\;\theta \right]}\\ & =\sqrt{{\left(b.x_{pr}\left(t\right)\right)}^{2}+{\left(g.x_{ch}\left(t\right)\right)}^{2}-2.g.b.x_{pr}\left(t\right)\times x_{ch}\left(t\right)\text{cos}\;\theta } \end{array}$$

Applying the value of |*b*.*x*_*pr*_(*t*) − *g*.*x*_*ch*_(*t*)|, in above Eq (15) that can be stated as in Eq (16).


$$x_{ch}\left(t+1\right)=x_{pr}\left(t\right)-c.\sqrt{{\left(b.x_{pr}\left(t\right)\right)}^{2}+{\left(g.x_{ch}\left(t\right)\right)}^{2}-2.g.b.x_{pr}\left(t\right)\times x_{ch}\left(t\right)\text{cos}\;\theta }$$ (16)

Squaring on both sides, we get the formulation as in Eq (17).


$$\begin{array}{l} {\left(x_{ch}\left(t+1\right)\right)}^{2}={\left(x_{pr}\left(t\right)\right)}^{2}-c^{2}\left[{\left(b.x_{pr}\left(t\right)\right)}^{2}+{\left(g.x_{ch}\left(t\right)\right)}^{2}-2.g.b.x_{pr}\left(t\right)\times x_{ch}\left(t\right)\text{cos}\;\theta \right]\\ {\left(x_{ch}\left(t+1\right)\right)}^{2}={\left(x_{pr}\left(t\right)\right)}^{2}-{\left(c.b.x_{pr}\left(t\right)\right)}^{2}-{\left(c.g.x_{ch}\left(t\right)\right)}^{2}+c^{2}.2.g.b.x_{pr}\left(t\right)\times x_{ch}\left(t\right)\text{cos}\;\theta \end{array}$$ (17)

Again squaring on both sides, we get the formulation as in Eq (18)

$$x_{ch}\left(t+1\right)=x_{pr}\left(t\right)-c.b.x_{pr}\left(t\right)-c.g.x_{ch}\left(t\right)+c.\sqrt{2.g.b.x_{pr}\left(t\right)\times x_{ch}\left(t\right)\text{cos}\;\theta }$$ (18)

Then the POA [22] is updated with this formulation to converge the optimal points in the search space. Then the position of the pelicans at the time of hunting can be stated as in Eq (19).


$$x_{i,j}^{P_{2}}=x_{i,j}+R.\left(1-\frac{t}{T}\right).\left(2rand-1\right).x_{i,j}$$ (19)

By adding the Eqs (18) and (19), we get the formulation as in Eq (20). Thereby, the above Eq (14) is modified with this derived new position formulation.


$$x_{ch}\left(t+1\right)=\left[\frac{x_{pr}\left(t\right)\left[1-c.b\right]+x_{ch}\left(t\right)\left[1+R\left(1-\frac{t}{T}\right)\left(2.rand-1\right)-c.g\right]+c.\sqrt{2.g.b.x_{pr}\left(t\right)\times x_{ch}\left(t\right)\text{cos}\;\theta }}{2}\right]$$ (20)

*3*.*7*.*1*.*2 Attacking approach*. In this exploitation phase, the attacking process is employs with the chimps, attacker. The optimum position regarding information is not available in the search space. So, the prey regarding information are collected by the chimps (attacker, driver, barrier, and chaser) and are stored to update the position of prey based on the position of best chimps that can be stated as in Eq (21). Here, *x*_*att*_ specifies position of attacker, *x*_*bar*_ specifies position of barrier, *x*_*cha*_ specifies position of chaser and *x*_*dri*_ specifies position of driver.


$$\begin{array}{l} D_{att}=\left|b_{1}x_{att}-g_{1}x\right|,\\ D_{bar}=\left|b_{2}x_{bar}-g_{2}x\right|,\\ D_{cha}=\left|b_{3}x_{cha}-g_{3}x\right|,\\ D_{dri}=\left|b_{4}x_{dri}-g_{4}x\right|. \end{array}$$ (21)

Then the relation between the chimps (attacker, driver, barrier, and chaser) can be stated as in Eq (22).


$$x\left(t+1\right)=\frac{x_{1}+x_{2}+x_{3}+x_{4}}{4}$$ (22)

*3*.*7*.*1*.*3 Prey attacking*. In the utilization phase, the prey attacking process is deploys that attacks the prey when the prey halt the motion. In this process, the *f* value is lowered by then, the *c* value is also lowered. Based on this operation, the location of the chimp is updated in accordance with the location of driver, barrier, chaser, and attacker. Yet, the proposed PUCOA hybrid optimization faces a challenge in trapping local minima. To overcome this issue, the exploration phase is employs that requires additional operators.

*3*.*7*.*1*.*4 Searching for prey*. In the "Searching for prey" process, the search approach is performed with consideration of the positions of the driver, barrier, attacker, and chaser to explore and locate the prey. The objective is to effectively search for and attack the prey while avoiding getting trapped in local minima. Initially, the search agents, representing the driver, barrier, attacker, and chaser, are spread across the search space. The search space refers to the area where the prey is expected to be located. By spreading the search agents across the search space, they increase the chances of locating the prey. The divergence model is designed to guide the search agents in their exploration. It utilizes a vector, which provides a direction for the search agents to follow. Additionally, random numbers greater than 1 or lower than -1 are employed to enhance exploration and prevent the agents from being stuck in local minima. By spreading out and exploring the search space while following the divergence model, the search agents can effectively locate the prey and converge towards it. This approach improves the optimization process by reducing the likelihood of getting trapped in local minima, which are suboptimal solutions. Overall, the combination of spreading the search agents, utilizing the divergence model with a vector and random numbers, and avoiding local minima traps helps enhance the effectiveness of the search process for locating and attacking the prey.

*3*.*7*.*1*.*5 Social incentive*. In this final phase, the chimps are subsequently involved in the sexual motivation and stop the responsibility of hunting process. This chaotic behaviour resolves the high dimensional issues by entrapping local minima and slow convergence rate. At the time of optimization process, the location of chimps is updated with the chaotic model that can be stated as in Eq (23). Here, *μ* specifies the random number in the interval [0, 1].


$$x_{ch}\left(t+1\right)=\left\{\begin{array}{ll} x_{pr}\left(t\right)-c.D & if\;\mu <0.5\\ Chaotic\_value & if\;\mu >0.5 \end{array}\right.$$ (23)

#### 3.7.2 Pseudo-code of proposed PUCOA

**Algorithm 1**: Proposed PUCOA algorithm

Initialize the population *x*_*i*_

Set *f*, *g*, *c and b*

Evaluate each chimp position

Split the chimps into groups randomly

**Until the** halting function is satisfied

Evaluate the fitness of each chimp

*x*_*att*_ = optimal search agent

*x*_*cha*_ = second optimal search agent

*x*_*bar*_ = third optimal search agent

*x*_*dri*_ = final optimal search agent

 **While** (t< max no. Of iterations)

  **For** each chimp:

   Retrieve the chimps’ community

   Group strategy is utilized to modify *f*, *g*, *and b*

   Deploys *f*, *g*, *and b* to evaluate *c* then *D*.

  **end for**

   **For** each search chimp

    if (*μ* < 0.5)

     if(|*a*| < 1)

     The new position of the present search agent is updated

     else if (|*a*| > 1)

     Choose the random search agent

     end if

    else if (*μ* > 0.5)

     The new position of the present search is updated

    end if

   **end for**

  Modify *f*, *g*, *c and b*

  Update *x*_*att*_ *x*_*cha*_ *x*_*bar*_ *x*_*dri*_

  T = t+1

 **end while**

**Return** *x*_*att*_

## 4. Proposed secured data transmission via improved blowfish algorithm

### 4.1 Secured data transmission

The data packets are transmitted from node to node that is of any kind. During the data transmission, security of the data is ensured by the improved cryptosystem. Thereby, this paper proposes improved blow fish cryptosystem to encrypt the original data.

### 4.2 Improved blow fish algorithm

The Improved Blowfish algorithm is a modified version of the original Blowfish algorithm, designed to address specific requirements and challenges in the context of UASN communication.

The key differences between the Improved Blowfish algorithm and the original Blowfish algorithm can include:

1. Security Enhancements: The Improved Blowfish algorithm may incorporate additional security features or modifications to enhance the encryption strength and resilience against potential attacks specific to UASN environments. These enhancements could include adjustments to the key generation, encryption/decryption processes, or the key schedule algorithm.
2. Performance Optimization: The Improved Blowfish algorithm may introduce optimizations to improve the encryption/decryption speed and efficiency, considering the resource constraints and computational limitations of UASN nodes. These optimizations aim to minimize the computational overhead and energy consumption while maintaining a high level of security.
3. Adaptability to UASN Requirements: The Improved Blowfish algorithm could be tailored to address the unique characteristics and challenges of UASN, such as the time-varying and space-varying nature of the underwater acoustic channel. It may incorporate techniques to mitigate the impact of channel variations on data transmission, ensuring reliable and secure communication in UASN deployments.
4. Customization for UASN-specific Constraints: The Improved Blowfish algorithm might be designed with considerations for the energy constraints, latency requirements, and bandwidth limitations of UASN. It could optimize the encryption/decryption operations to minimize energy consumption and reduce transmission delays, allowing efficient data transmission in resource-constrained underwater environments.

Blowfish algorithm [21] encrypts the original data of 64-bits with the dimension 1000 × 14 and it tracks the fetal network. It is a symmetric block cipher algorithm and the entire process splits into two sections: they are key expansion and data encryption that are described as follows:

#### 4.2.1 Key expansion

The key with 448 bits gets divided into numerous sub-key arrays in this section, such that the overall key is 4168 bytes. The steps to generate sub keys are as follows:

Step 1: First initialize the P-array. Then four S-boxes are initialized in form with a fixed string. This fixed string comprises of hexadecimal digits of pi.
Step 2: For all bits of the key are XOR with their relevant bits. With the initial 32 bits of key, the XOR P1 is getting operated. Likewise, the 32 bit keys are operated up to with XOR P14. This process gets repeated up to the whole P-array has been XOR with their key bits.

#### 4.2.2 Data encryption

In this section, the network can be iterated to 16 times and the available keys are in accordance with the key dependent permutation as well as the data and key dependent substitution, for every round. In this algorithm, the main operations involved are addition on 32- bit words or XOR operations. In the key generation process, blow fish utilizes numerous number of sub keys and these keys are already created to either data decryption or data encryption. The p-array comprises of 18 (i.e. P1, P2….P18) with 32- bit sub keys. Each four 32-bit S-boxes involve 256 entries that are represented as follows:

S-box 1: S1, 0, S1, 1…S1, 255
S-box 2: S2, 0, S2, 1…S2, 255
S-box 3: S3, 0, S3, 1…S3, 255
S-box 4: S4, 0, S4, 1…S4, 255

#### 4.2.3 Function F

The function F of blow fish algorithm is improved in this work. Then the improved function F can be stated as in Eq (24).


$$\begin{array}{ll} F & =\left\{\left(S_{2}\left[a\right]+S_{3}[a]+\left(S_{4}\left[a\right]>>1\right)\text{mod}\;2^{23}\right)XOR\right.\\ & \left((S_{1}\left[a\right]<<1)+S_{3}[a]+S_{4}\left[a\right]\text{mod}\;2^{23}\right)XOR\\ & \left(S_{1}\left[a\right]+(S_{2}\left[a\right]>>1)+S_{4}\left[a\right]\text{mod}\;2^{23}\right)XOR\\ & \left(S_{1}\left[a\right]+S_{2}\left[a\right]+(S_{3}[a]>>1)\text{mod}\;2^{23}\right)XOR\} \end{array}$$ (24)

**Fig 4:**
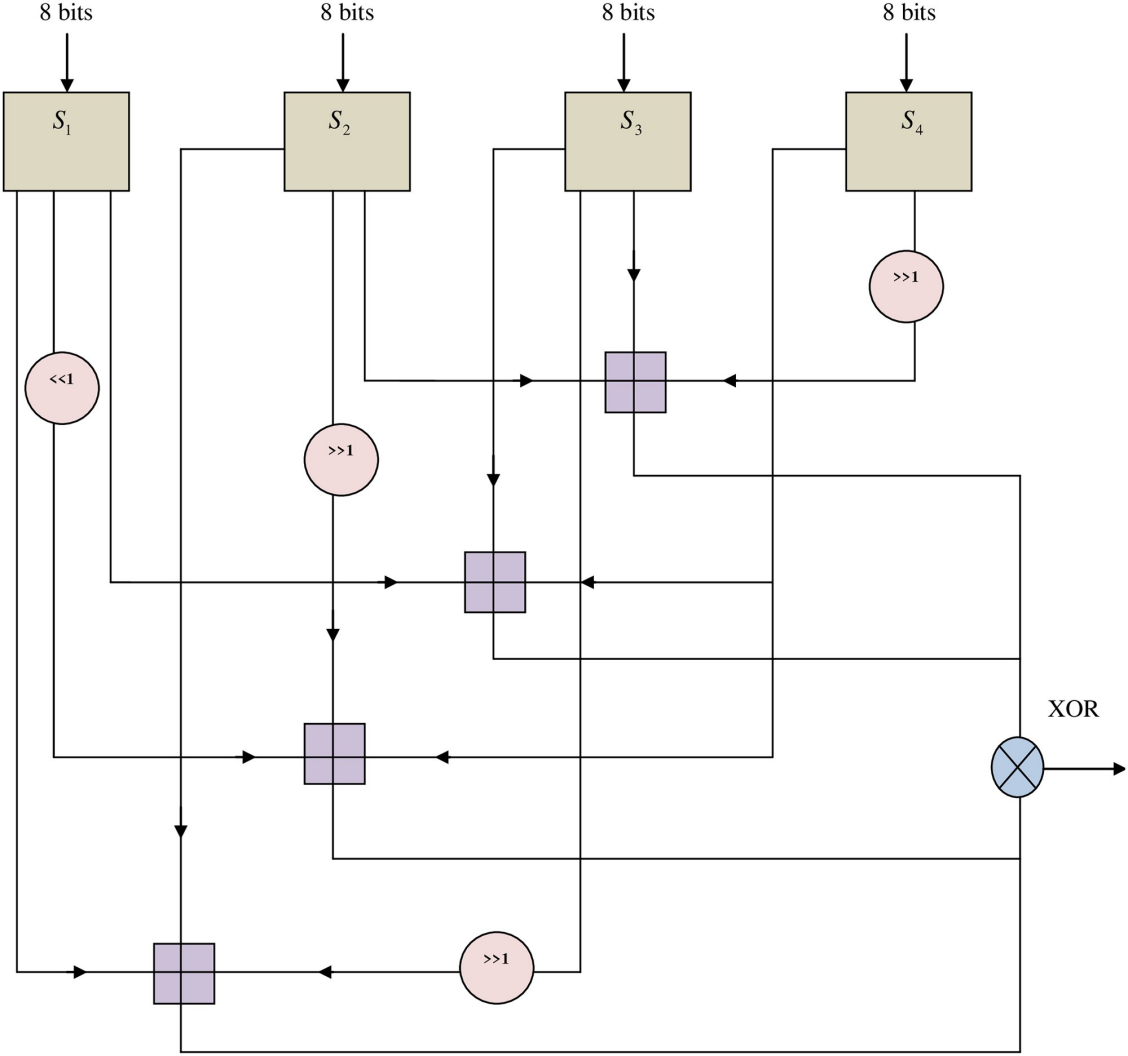
The process of function F in the improved Blowfish algorithm.

The work flow of function F has depicted in the below “Fig 4”. The “Fig 4” depicts the implementation of new function F in the improved Blow fish. It includes four S-boxes, the first S-box i.e. *S*_1_ allotted with first 8 bits, the next S-box i.e. *S*_2_ allotted with next 8 bits, and so on up to the fourth S-box (*S*_4_). When each 8 bit data is entered into each S-box, the bit value is modified into 32-bit value. During this process, before the value is inputted into the S-box, several variables are transformed to the left or right side. The modified 32-bit value created in the *S*_4_ is left shifted and XOR with the *S*_2_ and *S*_3_. Likewise, the remaining S-boxes can perform the same procedure. Finally, the output of 64 bit value was obtained by concatenating the values from *S*_1_, *S*_2_, *S*_3_, and *S*_4_ and the data is decrypted at the receiver side. Thus, the secured data is transmitted though the optimal path.

## 5. Results and discussion

### 5.1 Simulation procedure

The proposed methodology based on secure data transmission via optimal path selection was implemented in MATLAB. In order to assess the accurate prediction of energy, the LSTM is compared over the existing classifiers like Artificial Neural Network (ANN), Recurrent Neural Network (RNN), Deep Belief Network (DBN), Gated Recurrent Unit (GRU) and Convolutional Neural Network (CNN) in terms of Precision, False Detection Rate (FDR), False Negative Rate (FNR), False Positive Rate (FPR), Accuracy, Matthews Correlation Coefficient and other measures. Additionally, for the optimal path selection, the analysis was carried out about Energy, Link Quality and Distance as well as the PUCOA is contrasted with the conventional optimization methods. Furthermore, the attack analysis (Chosen-Ciphertext Attack (CCA) and Chosen-plaintext Attack (CPA)) was conducted to evaluate the secure transmission of data and it is examined over the traditional encryption algorithms (BlowFish Algorithm (BF), Rivest-Shamir-Adleman (RSA) and Elliptical Curve Cryptography (ECC)) with Improved BlowFish Algorithm (IBFA)

### 5.2 Assessment on positive metric of the LSTM and the existing systems for energy prediction

**Fig 5:**
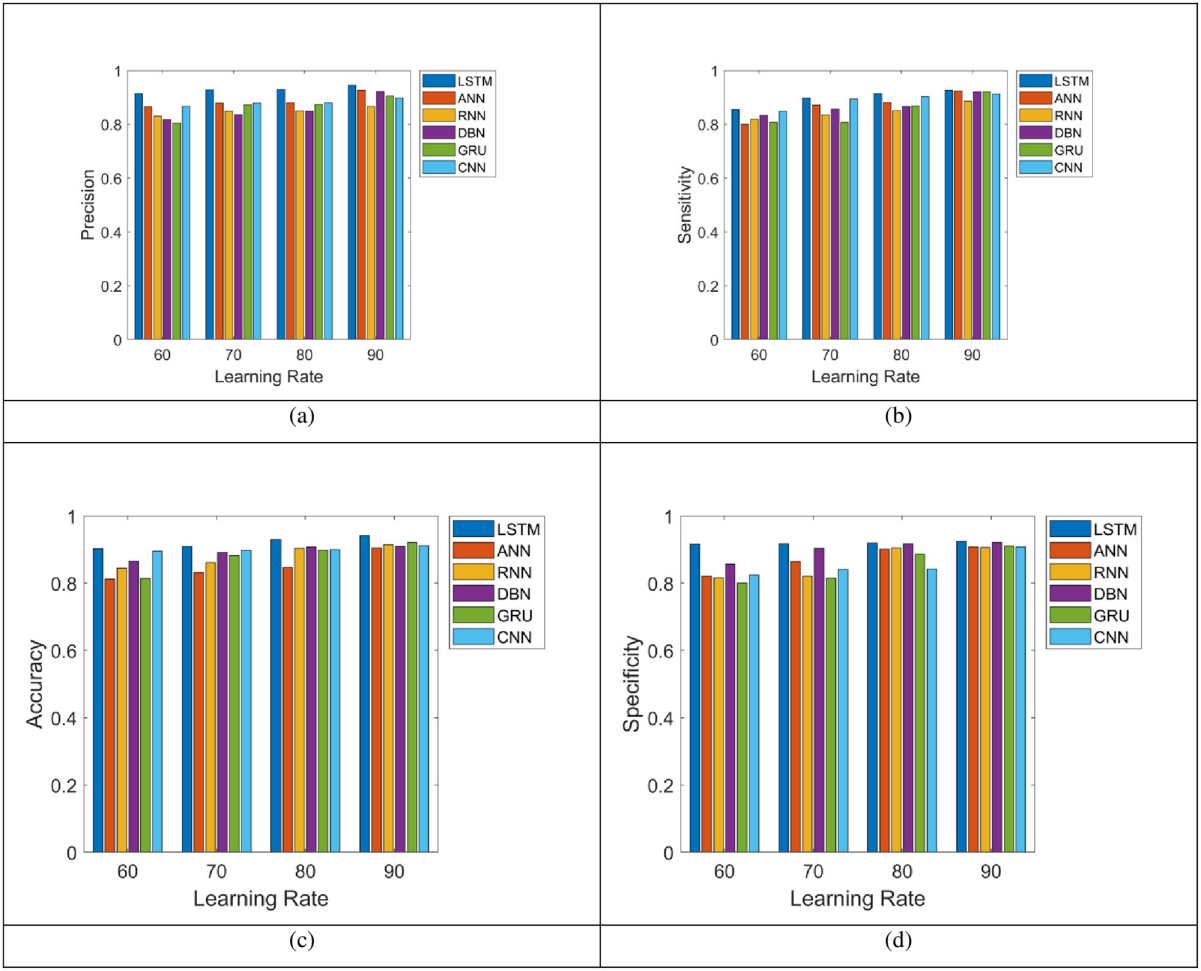
Energy prediction analysis on LSTM and the conventional techniques using the positive metric.

“Fig 5” shows the positive measure evaluation on LSTM is contrasted over the ANN, RNN, DBN, GRU and CNN for the prediction of energy. The examination is conducted for varied number of learning rate. For the prediction of energy, the positive measures need to be higher. Based on the “Fig 5(c)”, the LSTM generated the highest accuracy (93.78%) for the learning rate 80% with better prediction of energy, though the ANN, RNN, RNN, DBN, GRU and CNN have gained the least accuracy value. Furthermore, the precision obtained by the LSTM is 95.38%, meanwhile the ANN is 90.78%, RNN is 83.43%, DBN is 82.72%, GRU is 85.96% and CNN is 87.63%, correspondingly, based on the “Fig 5(a)”.

Regarding the “Fig 5(b)”, while the learning rate is 70%, the LSTM model has attained better sensitivity; still, the extant methods (ANN, RNN, DBN, GRU and CNN) attained very lower specificity. In addition, the specificity of LSTM attain larger performance in learning percentage 90% is 95.85% for the prediction of energy, whereas the conventional methods scored the least specificity, including, ANN = 84.65%, RNN = 82.94%, DBN = 85.63%, GRU = 83.54% and CNN = 87.65%, respectively as shown in “Fig 5(d)”. As shown by the positive metric analysis, the LSTM improves the accuracy level and it is capable to predict the energy.

### 5.3 Assessment on negative metric of the LSTM and the existing systems for energy prediction

**Fig 6:**
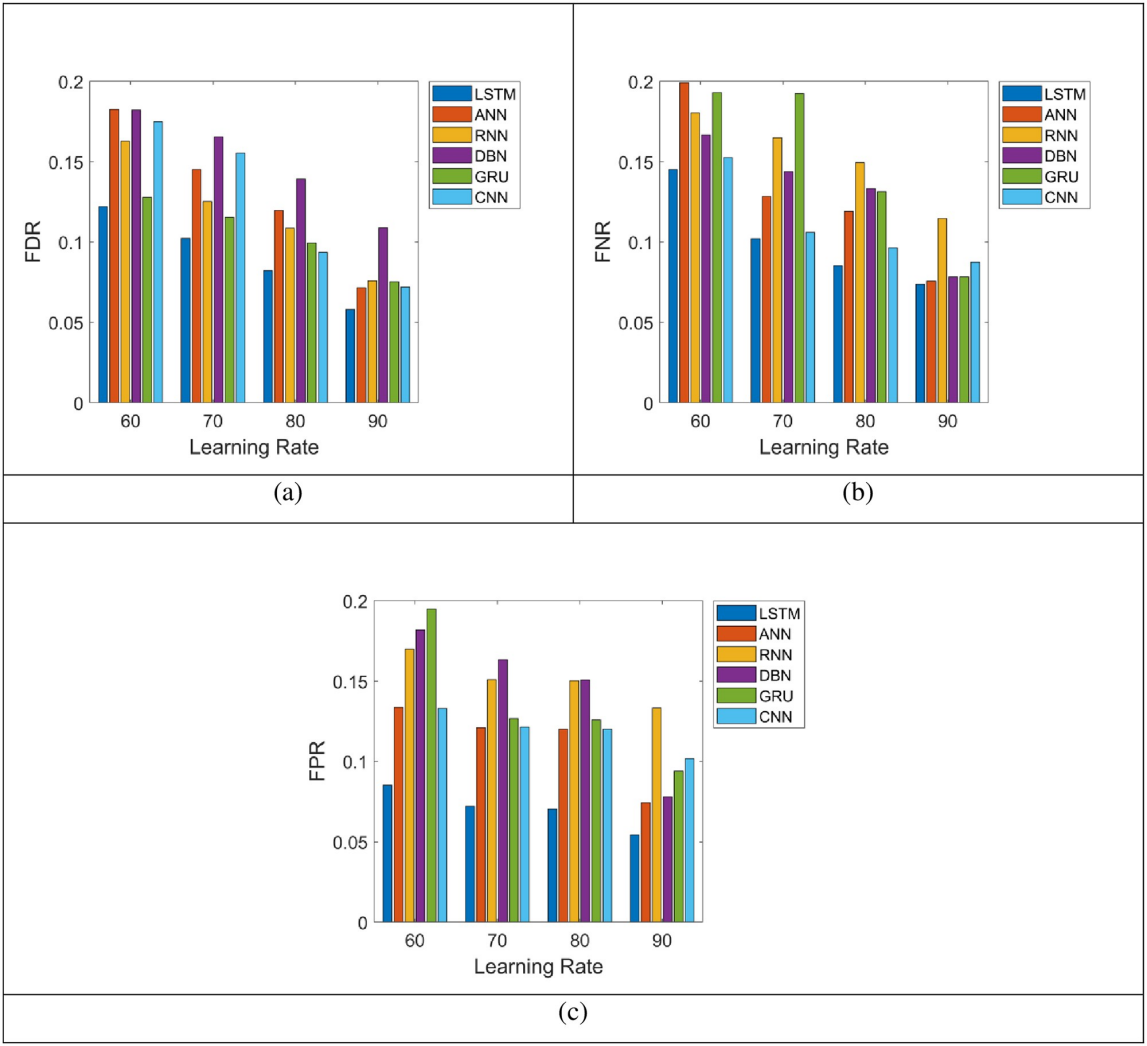
Energy prediction analysis on LSTM and the conventional techniques using the negative metric.

The analysis on LSTM over the conventional methods like ANN, RNN, DBN, GRU and CNN regarding negative measure for the prediction of energy is exposed in “Fig 6”. Further, the LSTM offered the least negative metric values with accurate prediction of energy than the established methods. More particularly, the FDR of LSTM in the learning rate 80% is 0.053, this is superior to ANN (0.074), RNN (0.083), DBN (0.124), GRU (0.081) and CNN (0.076), correspondingly shown in “Fig 6(a)”. The LSTM approach holds minimum FNR of approximately 0.065 for the training rate 90% as per “Fig 6(b)” than other prior schemes like ANN, RNN, DBN, GRU and CNN. Moreover, the FPR of the LSTM acquires minimal value for learning rate 90% with better energy prediction when compared to the FPR rate for learning rate 60% is displayed in “Fig 6(c)”. Therefore, the LSTM has achieved improved performance with fewer negative metric values with accurate prediction of energy.

### 5.4 Assessment on other metric of the LSTM and the existing systems for energy prediction

**Fig 7:**
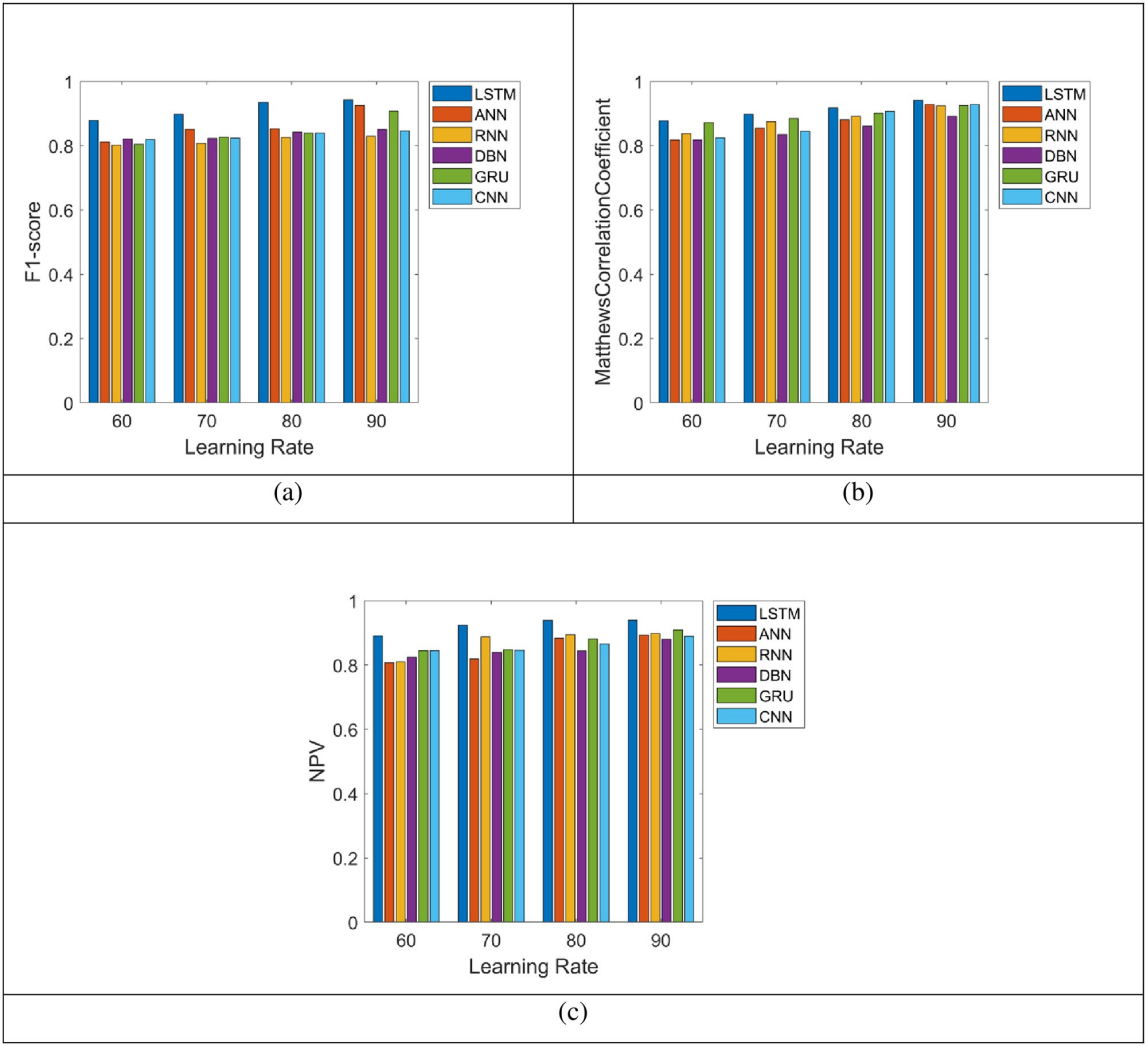
Energy prediction analysis on LSTM and the conventional techniques using the other metric.

“Fig 7” depicts the other measure assessment on LSTM over the ANN, RNN, DBN, GRU and CNN for the prediction of energy in terms of other measures (F1-score, Matthews Correlation Coefficient and Negative Predictive Value (NPV)). Likewise, the F1-score of the LSTM for the learning rate 60 in “Fig 7(a)” is approximately 90.84%, whereas the ANN, RNN, DBN, GRU and CNN recorded the least F1-score ratings. Additionally, the LSTM offered the highest Matthews Correlation Coefficient of 96.79% at the training rate 90% and the conventional strategies gained the minimized correlation coefficient ratings, such as, ANN = 87.21%, RNN = 88.76%, DBN = 83.64%, GRU = 86.74% and CNN = 89.97%, respectively as shown in “Fig 7(b)”. Consequently, the NPV of the LSTM is much higher with accurate prediction of energy in almost all the learning rates. The entire evaluation makes it clear that the LSTM approach is substantially more successful at forecasting the energy as shown in “Fig 7(c)”.

### 5.5 Analysis on PUCOA and the conventional methods with respect to distance, energy consumption and link quality for optimal path selection

**Fig 8:**
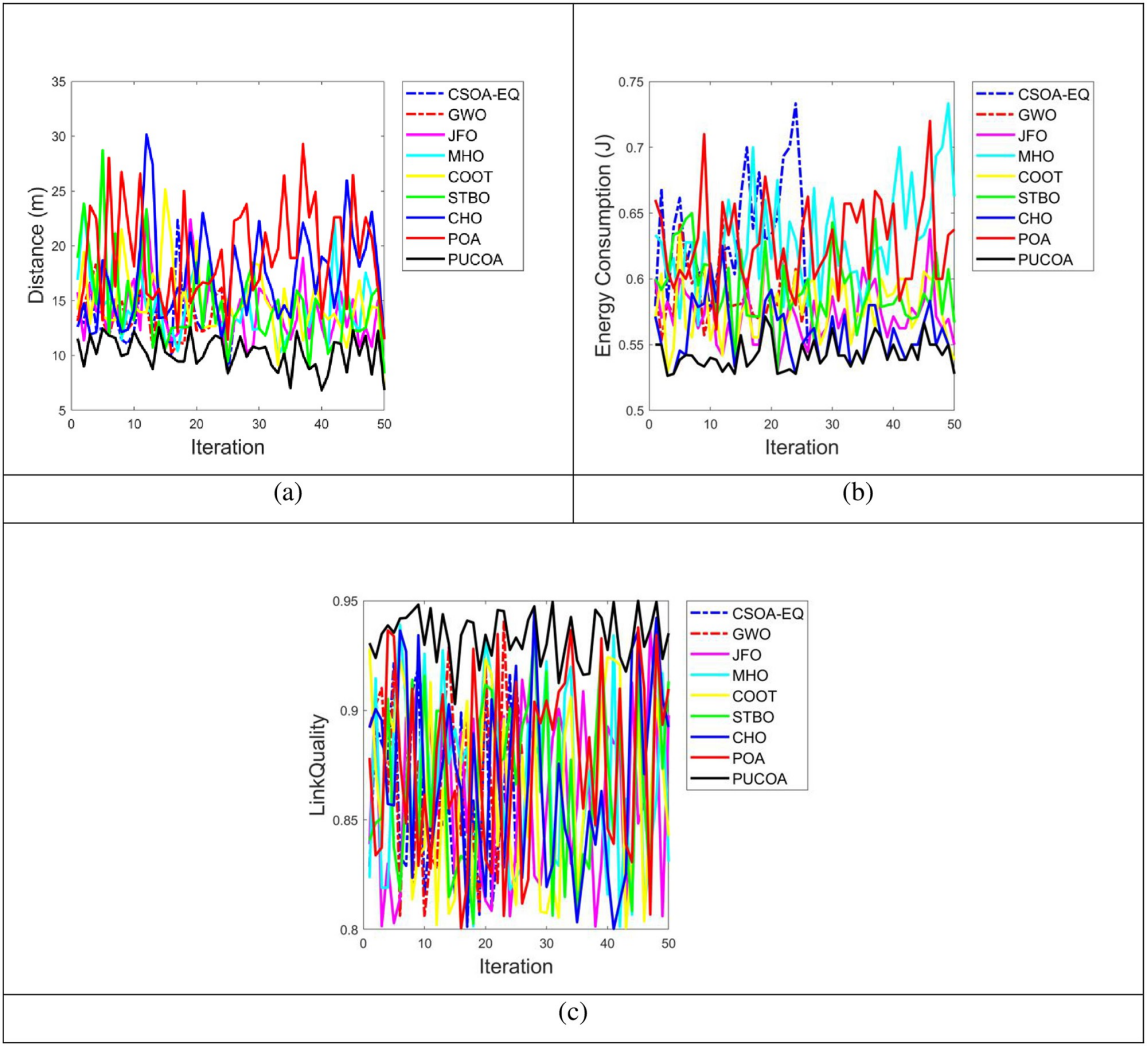
Examination on PUCOA and the traditional approaches for optimal path selection a) Distance b) Energy Consumption and c) Link Quality.

The PUCOA is compared over the Cuckoo Search Optimization Algorithm with Energy Efficient and QOS Aware (CSOA-EQ) [6], Grey Wolf Optimization Algorithm (GWO) [2], Jelly Fish Optimization (JFO), Marriage Honeybee Optimization (MHO), COOT Optimization (COOT), Sewing Training Based Optimization (STBO), CHO [23] and POA [22] in terms of distance, energy consumption and link quality for choosing the optimal path is represented in “Fig 8(a)” to “Fig 8(c)”. To choose the optimal path, it is necessary to reduce the distance and energy use. In a similar way, the PUCOA consumed lesser energy with minimized distance. More particularly, the 50th iteration, the PUCOA achieved (8m) shortest distance score than the CSOA-EQ, GWO, JFO, MHO, COOT, STBO, CHO and POA, correspondingly. Moreover, the energy consumed by the PUCOA in the iteration 40 is 0.53J with optimal path selection, even though the CSOA-EQ, GWO, JFO, MHO, COOT, STBO, CHO and POA have consumed highest amount of energy. Further, the link quality must be high for the effective performance of the system. The PUCOA method outperformed the current techniques from the first to the last iteration in terms of link quality rate. For instance, the PUCOA attained the greatest link quality rate of 0.95 for the 30th iteration with choosing the optimal path over the CSOA-EQ, GWO, JFO, MHO, COOT, STBO, CHO and POA. Thus, the optimal path is selected, guaranteeing that sensitive data is secured as well. This improvement is made possible by the hybrid optimization method.

### 5.6 Evaluation on IBFA and the traditional approaches with regard to CCA, CPA attack analysis and key sensitivity for secure data transmission

**Fig 9:**
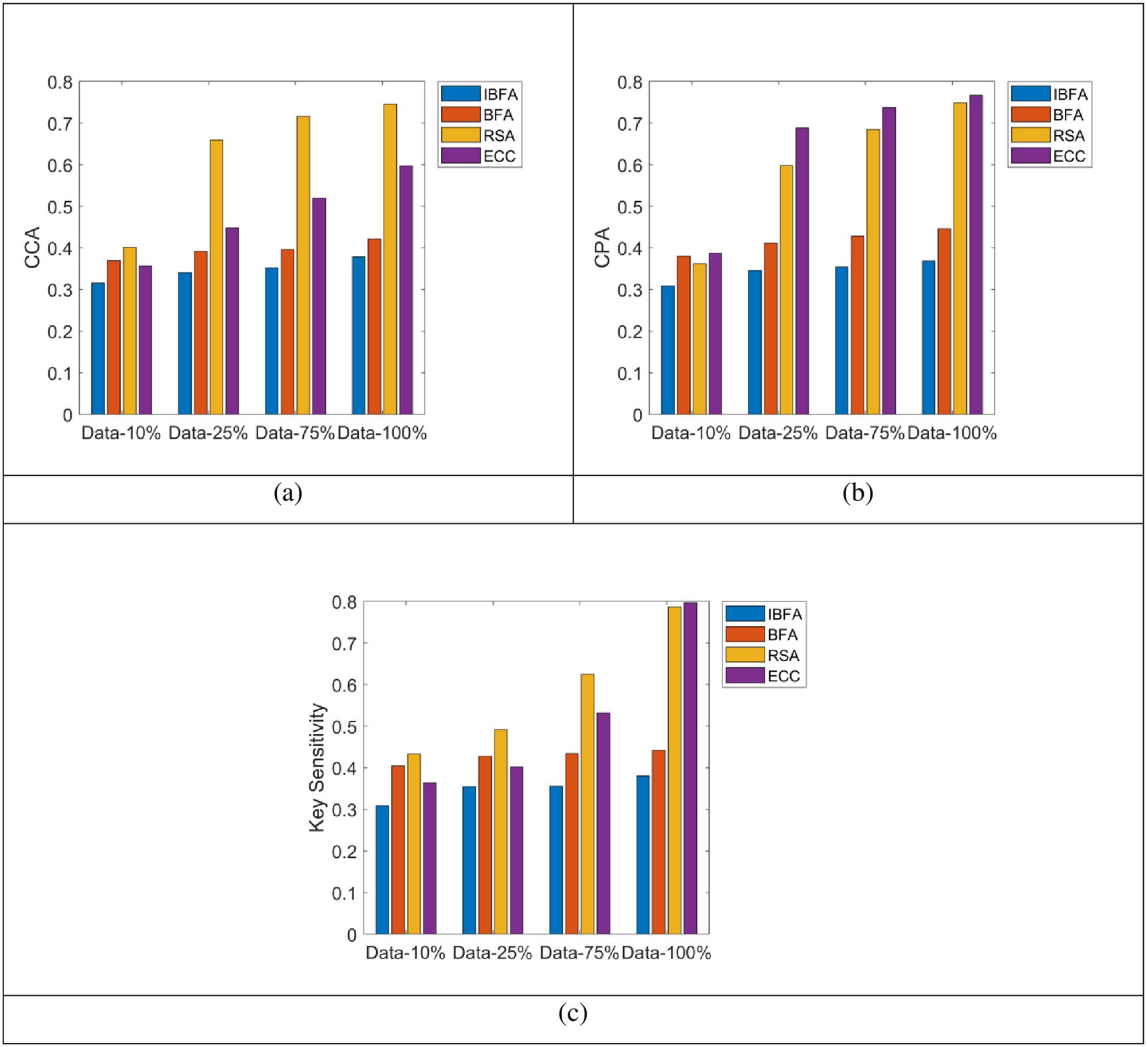
Examination on IBFA and the traditional approaches for secure data transmission a) CCA attack b) CPA attack and c) Key Sensitivity.

“Fig 9(a)” and “9(b)” displays the CCA and CPA attack analysis on IBFA over the traditional encryption methods like BF, RSA and ECC for secure data transmission. Also, it is examined by transmitting the data in increments of 10%, 25%, 75%, and 100%. Moreover, the CCA attack is referred to as, “An attack strategy on cryptanalysis known as a chosen-ciphertext attack (CCA) allows the cryptanalyst to collect data via acquiring the decryptions of specified ciphertexts.” The description states that an approach with a lower value is more secure. Additionally, the IBFA has the lowest CCA attack rating up to 100% of data transfer. Furthermore, when the 10% of data transmitted, the IBFA recorded the least attack ratings of 0.3, though the BF (0.36), RSA (0.39) and ECC (0.38) scored the highest attack value. In addition, the CPA is said to as, “A chosen-plaintext attack (CPA) is indeed a type of cryptanalysis attack which assumes the attacker has access to the plaintext ciphers for every given plaintext. The attack’s objective is to obtain data that will render the encryption system less secure.” In a similar manner, the IBFA method effectively lowered the CPA attack rate during 100% of data transfer than the BF, RSA, and ECC. In this section, it is apparent that the IBFA could provide data transmission with absurdly high levels of safety.

Finally, analyzing the outcomes of the IBFA over the tradition methods in terms of key sensitivity analysis for secure data transmission is portrayed in “Fig 9(c)”. Mainly, the correlation coefficient of the IBFA for the 25% of data transmission is 0.35, whereas the BF is 0.43, RSA is 0.49 and ECC is 0.38, respectively. It implies that the IBFA ensure the protected data preservation due to the difficulty of identifying the original data without the appropriate key.

### 5.7 Convergence graph assessment on PUCOA and the conventional methods for secure data transmission via optimal path selection

**Fig 10:**
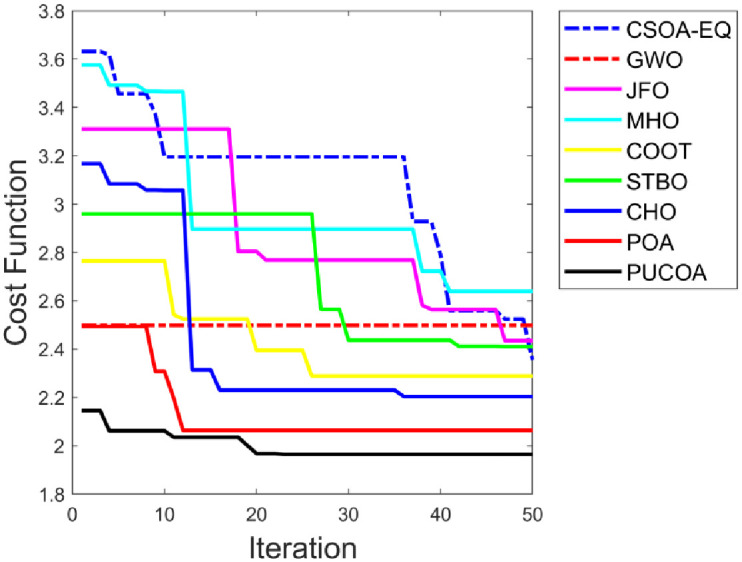
Convergence study on PUCOA versus conventional techniques for secure data transmission via optimal path selection.

The cost analysis of the PUCOA is contrasted to the CSOA-EQ, GWO, JFO, MHO, COOT, STBO, CHO and POA is illustrated in “Fig 10”. In addition, the evaluation is conducted for distinct number of iterations (0–50). In order to choose the optimal path, the cost rate must be low with faster convergence. The PUCOA received the lowest cost value from the first (0th) to the last (50th) iterations with quick convergence. Further, the PUCOA scored the cost value of 2.19 in the 0th iteration and it gets lowered from the iteration 20 to 50 as well as it achieved the minimal cost rate of 1.94. Moreover, the worst cost rate is provided by the MHO is 2.63, GWO is 2.54 and JFO is 2.47, respectively. As a result, the PUCOA outperformed previous approaches admirably, proving its greater capacity to safeguard data during transmission.

### 5.8 Computational time analysis on proposed over the conventional techniques for secure data transmission via optimal path selection

Table 2 summarizes the computational time evaluation on IBFA over the BF, RSA and ECC for secure data transmission. It is analyzed under 10%, 25%, 75% and 100% of data transmission. For the 100% of data transmission, the IBFA obtained the lowest computational time of 23.616 and the current encryption methods scored the least computational time, including, BF = 25.681, RSA = 26.324 and ECC = 24.988. Additionally, the computation time assessment is conducted for the PUCOA over the POA, CHO, STBO, COOT, MHO, JFO, GWO and CSOA-EQ for the optimal path selection is shown in Table 3. More particularly, the PUCOA recorded the least computational time (13.307) over the POA, CHO, STBO, COOT, MHO, JFO, GWO and CSOA-EQ, correspondingly.

**Table 2:** Analysis on computational time for secure data transmission.

Methods	Data-10%	Data-25%	Data-75%	Data-100%
IBFA	15.166	18.141	18.662	23.616
BF	19.95	20.011	25.479	25.681
RSA	17.759	22.41	22.954	26.324
ECC	17.118	22.323	22.624	24.988

**Table 3:** Analysis on computational time for optimal path selection.

Methods	Computational Time(sec)
PUCOA	13.307
POA	14.187
CHO	21.488
STBO	24.334
COOT	30.007
MHO	28.338
JFO	31.694
GWO	17.577
CSOA-EQ	29.143

### 5.9 Statistical evaluation on IBFA and the conventional methods with regard to key sensitivity for secure data transmission

The statistical assessment on IBFA is compared over the traditional encryption methods like BF, RSA and ECC for secure transmission of data is displayed in Table 4. Also, it is evaluated in terms of key sensitivity for varied types of statistical measures. The key sensitivity must be lowered for the secure data transmission. More particularly, for the best statistical measure, the IBFA obtained the least correlation value of 0.3088, though the BF, RSA and ECC scored the highest correlation value of 0.4045, 0.4339 and 0.3640. Further, the correlation rate of the IBFA at the mean statistical measure is 0.3501, whilst the BF is 0.4272, RSA is 0.5844 and ECC is 0.5236, respectively. According to the statistical analysis report, the suggested has a lower correlation rate across all statistical measures and meets all the requirements for secure data transmission.

**Table 4:** Statistical study in terms of key sensitivity for secure data transmission.

Methods	Best	Standard Deviation	Median	Worst	Mean
IBFA	0.30889	0.029819	0.35567	0.38022	0.35011
BF	0.40454	0.016214	0.43115	0.44209	0.42723
RSA	0.43398	0.15678	0.5585	0.78665	0.58441
ECC	0.36403	0.19566	0.46707	0.79655	0.52368

### 5.10 Encryption and decryption time analysis on IBFA and the traditional encryption schemes for secure data transmission

The encryption and decryption time evaluation on IBFA over the conventional encryption methods, including, BF, RSA and ECC is illustrated in Tables 5 and 6. Additionally, the IBFA and the conventional methods are analyzed under 10%, 25%, 75% and 100% of data transmission. The encryption time and decryption time should be lowered for transmitting the data in a secure manner. Considering the Table 5, for the data transmission 100%, the IBFA recorded the lesser encryption time of 3.22, mean while the BF is 3.68, RSA is 3.38 and ECC is 3.60, respectively. In addition, the decryption time of the IBFA is 0.077 for the 10% of data transmission, which is much lower over the BF (0.702), RSA (1.027) and ECC (0.886). In conclusion, the IBFA based on secure data transmission produced outstanding results with enhanced performance in comparison of existing encryption methods.

**Table 5:** Encryption time analysis for secure data transmission.

Methods	Data-10%	Data-25%	Data-75%	Data-100%
IBFA	0.10061	0.23219	1.4081	3.2286
BF	0.12756	1.4996	2.6714	3.6885
RSA	1.2847	2.652	3.2298	3.385
ECC	0.48089	1.8675	2.4172	3.6006

**Table 6:** Decryption time analysis for secure data transmission.

Methods	Data-10%	Data-25%	Data-75%	Data-100%
IBFA	0.077779	1.3741	1.5555	2.8742
BF	0.70269	1.8832	2.5748	4.0725
RSA	1.027	1.585	2.7425	4.7752
ECC	0.88648	1.7287	2.607	4.6496

The performance of the proposed LSTM+PUCOA model is evaluated based on various metrics like accuracy, precision, specificity, sensitivity, FPR, F1 score, MCC, FNR, NPV, and FDR. And, these metrics helps to analyse the performance of the model for the given data. Particularly, the developed LSTM+PUCOA model is authenticated with standard benchmark models and it proves that the performance of the proposed LSTM+PUCOA model attains 90.85% of accuracy, 92.78% of precision, 91.78% of specificity, 89.79% of sensitivity, 7.21% of FPR, 89.76% of F1 score, 89.77% of MCC, 10.20% of FNR, 92.45% of NPV, and 10.22% of FDR for Learning percentage 70 which is represented in Table 7.

**Table 7:** Performance evaluation metrics.

Parameter (in %)	LSTM+PUCOA	ANN	RNN	DBN	GRU	CNN
Accuracy	0.90852	0.8313	0.86157	0.89157	0.88158	0.89816
Sensitivity	0.89793	0.8716	0.83513	0.85617	0.8076	0.894
Specificity	0.91781	0.86462	0.821	0.90393	0.81465	0.84124
Precision	0.92786	0.87896	0.84894	0.83653	0.87327	0.87857
FPR	0.072144	0.12104	0.15106	0.16347	0.12673	0.12143
F1-score	0.89763	0.85048	0.80721	0.82295	0.82667	0.82383
MCC	0.89772	0.85479	0.8746	0.83455	0.88447	0.8445
FNR	0.10207	0.1284	0.16487	0.14383	0.1924	0.106
NPV	0.92453	0.81843	0.88736	0.83888	0.84806	0.84626
FDR	0.10228	0.14521	0.1254	0.16545	0.11553	0.1555

## 6. Conclusion

This paper presents a novel model for optimal path selection and secure data transmission in UASN, utilising LSTM-based energy prediction. The proposed model comprises of two primary phases, namely node selection based on energy, distance, and link quality considerations, followed by the utilisation of the PUCOA algorithm to determine the optimal path. In addition, the data transmission is secured through an enhanced Blowfish encryption algorithm. The results of the experiment have shown the efficacy of the model we have proposed. The energy prediction using LSTM yielded a noteworthy F1-score of around 90.84% with a learning rate of 60, outperforming other methodologies including ANN, RNN, DBN, GRU, and CNN. The model that we have proposed offers a promising approach for selecting optimal paths and ensuring secure data transmission in UASN. The present research can be expanded by incorporating advanced optimisation techniques and novel variations of deep learning to address real-time challenges in this domain, particularly those related to link breakage in the path. Furthermore, there are plans to enhance the multi-objective constraints to enable a more precise selection of the optimal path. Additionally, there is a proposal to emphasise all of the recommended methodologies within the real-time system to enhance its flexibility.

## Supporting information

S1 File (ZIP)

## Abbreviations

ANN: Artificial Neural Network
ARQ: Automatic Repeat Request
CARQ: Cooperative ARQ
CNN: Convolutional Neural Networks
COA: Chimp Optimization Algorithm
CSOA-EQ: Cuckoo Search Optimization Algorithm with Energy Efficient and QOS Aware
DBN: Deep Belief Network
DBR: Depth-based Routing
DL-HDBT: Deep learning-based hybrid dynamic Biased track
EGRC: Energy-efficiency Grid Routing based on 3D Cubes
FEC: Forward Error Correction
GRU: Gated Recurrent Units
GWO: Grey Wolf Optimization Algorithm
HARQ: Hybrid automatic repeat request scheme
HDBT: Hybrid Dynamic Biased Track
LSTM: Long Short Term Memory
POA: Pelican Optimization Algorithm
PSO: Particle Swarm Optimization
PUCOA: Pelican Updated Chimp Optimization Algorithm
RNN: Recurrent Neural Network
TSDBG: Trust Strategy-based dynamic Bayesian Game
UASN: Underwater Acoustic Sensor Network
UASN: Underwater Acoustic Sensor Networks
(*W*_1_, *W*_2_ and *W*_3_): Weights coefficients
*a*: Frequency related variable
*A*(*x*): Function assisted to underwater acoustic propagation model
*A*_*i*_: Advanced nodes
*b*_*f*_: Bias vector of forget gate
*b*_*i*_: Bias vector of input gate
*b*_*o*_: Specifies bias vector of the output gate
*D*: Distance
*Dist*: Distance
*E*: Energy
*Fg*_*t*_: Forget gate
*h*_*t*−1_: Output state of the hidden layer
*Ig*_*t*_: Input gate
*I*_*i*_: Intermediate nodes
*k*: Parameter assisted to the underwater acoustic propagation model
*l*_*N*_: Location of the node *l*_*N*_
*Lq*: Link quality
*nE*: Node energy
*N*_*i*_: Normal nodes
*N*_*T*_: Node type
*Og*_*t*_: Output gate
*P*: Total number of packets
*P*_0_: Power
*P* ^*R*^: Packets received at each node
*S*: Sigmoid function
*S*_*d*_: Sending delay node
*t*: Current iteration set
tanh: Hyperbolic tangent function
*w*_*c*_: Weight of the candidate gate
*w*_*f*_: Weight of the forget gate
*w*_*i*_: Weight of the input gate
*w*_*o*_: Weight of the output gate
*x*_*at*_: Position of attacker
*x*_*bar*_: Position of barrier
*x*_*ch*_: Position of chaser
*x*_*ch*_: Position of chimp
*x*_*dri*_: Position of driver
*x*_*pr*_: Position of prey
