# Supplementary figures and images for: Optimal path selection and secured data transmission in underwater acoustic sensor networks: LSTM-based energy prediction
Source: PLoS One. 2023 Sep 5;18(9):e0289306. doi: 10.1371/journal.pone.0289306 (PMC10479917; doi:10.1371/journal.pone.0289306)

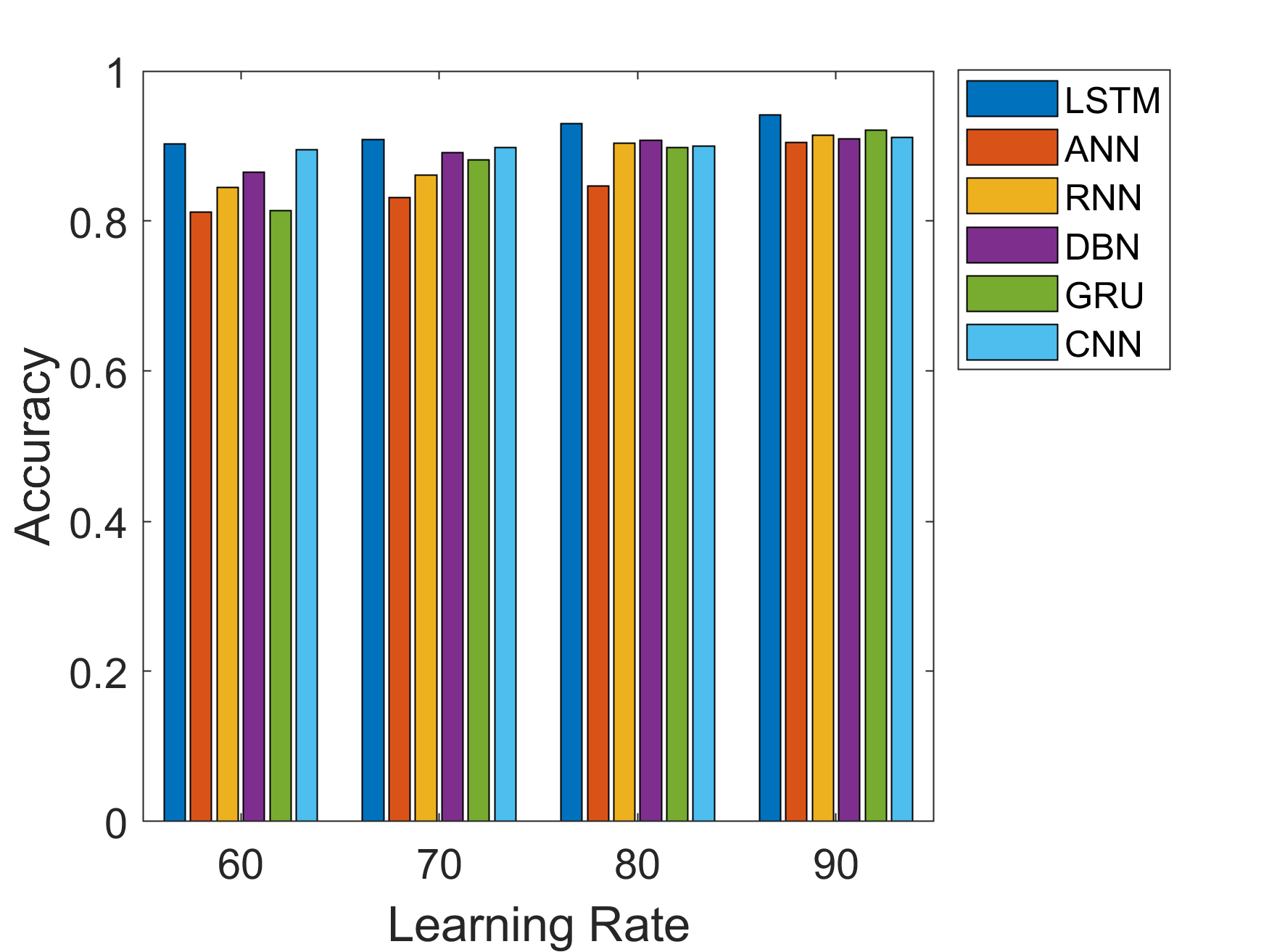

Supplement: S1 File — (ZIP) [file pone.0289306.s001.zip › Supporting Information files/Accuracy.tif]

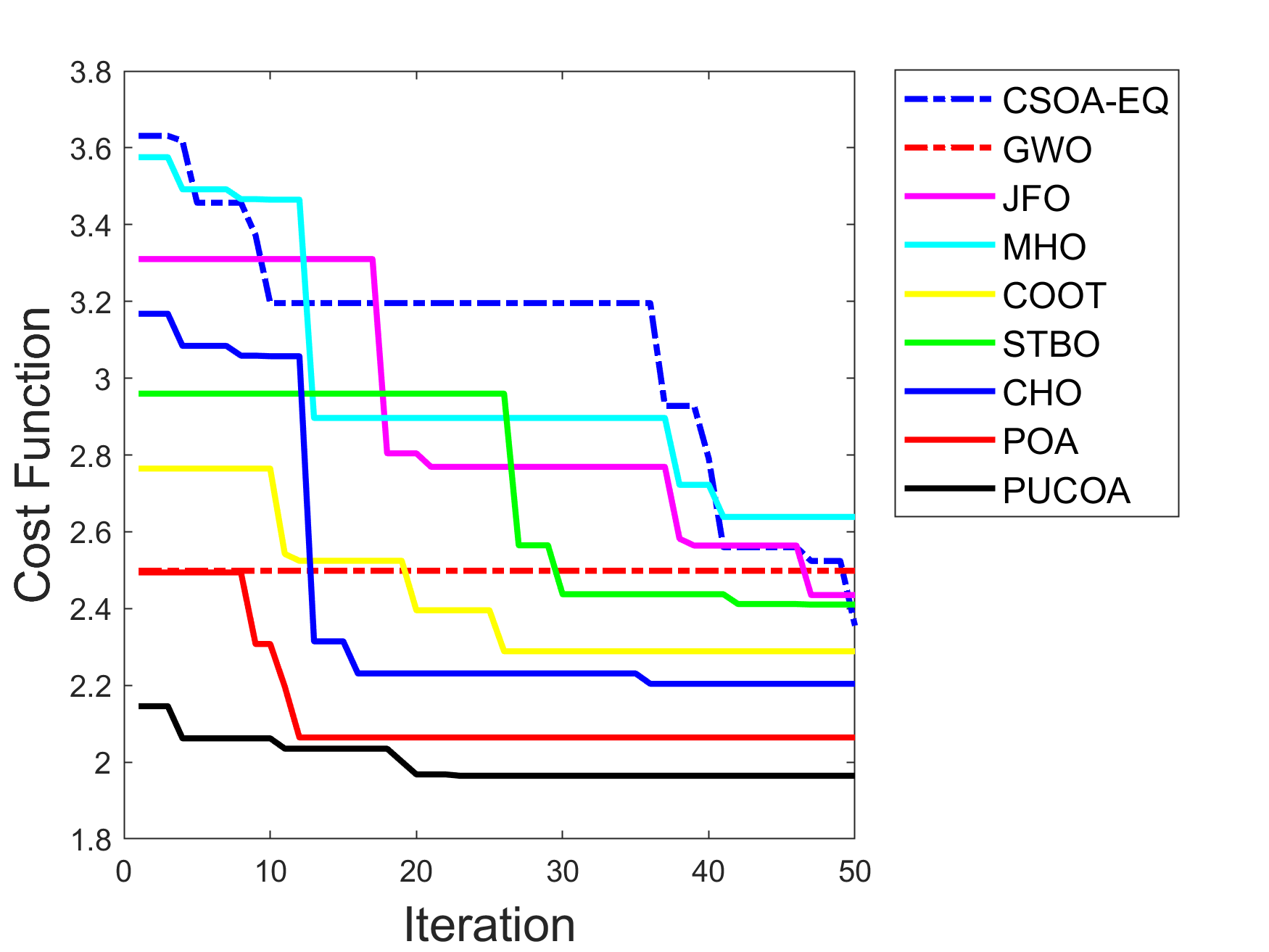

Supplement: S1 File — (ZIP) [file pone.0289306.s001.zip › Supporting Information files/convergence.tif]

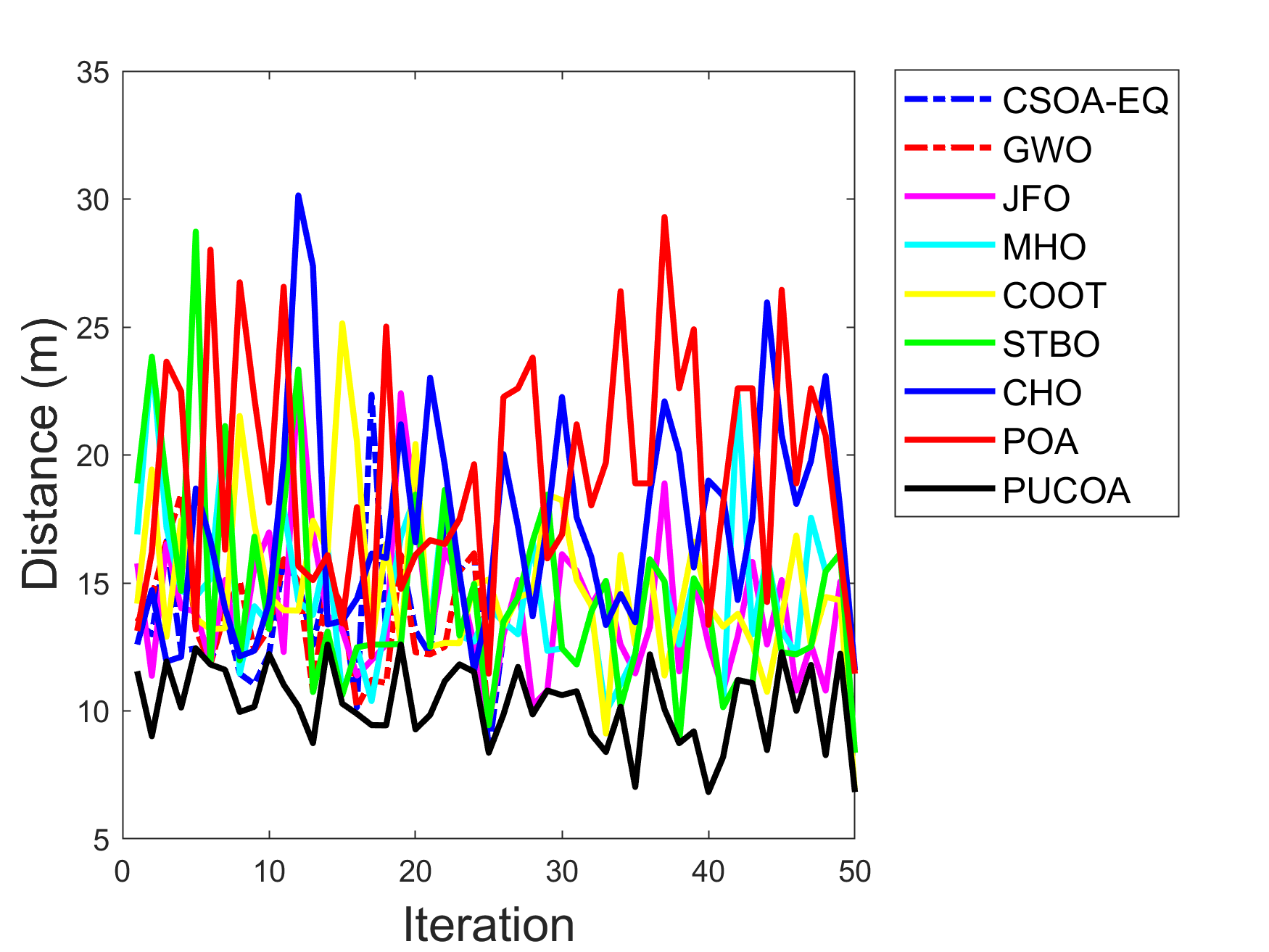

Supplement: S1 File — (ZIP) [file pone.0289306.s001.zip › Supporting Information files/Distance.tif]

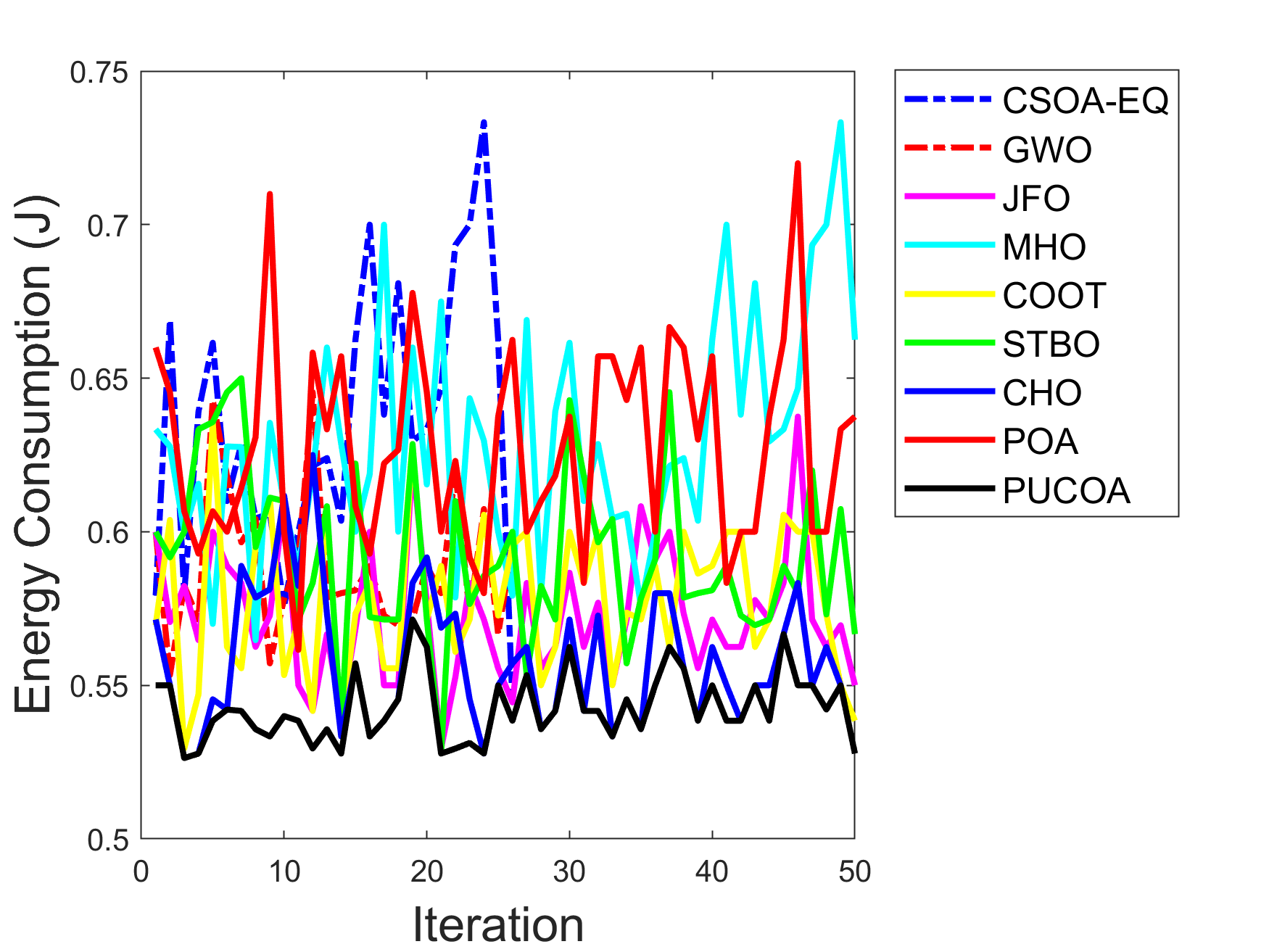

Supplement: S1 File — (ZIP) [file pone.0289306.s001.zip › Supporting Information files/Energy.tif]

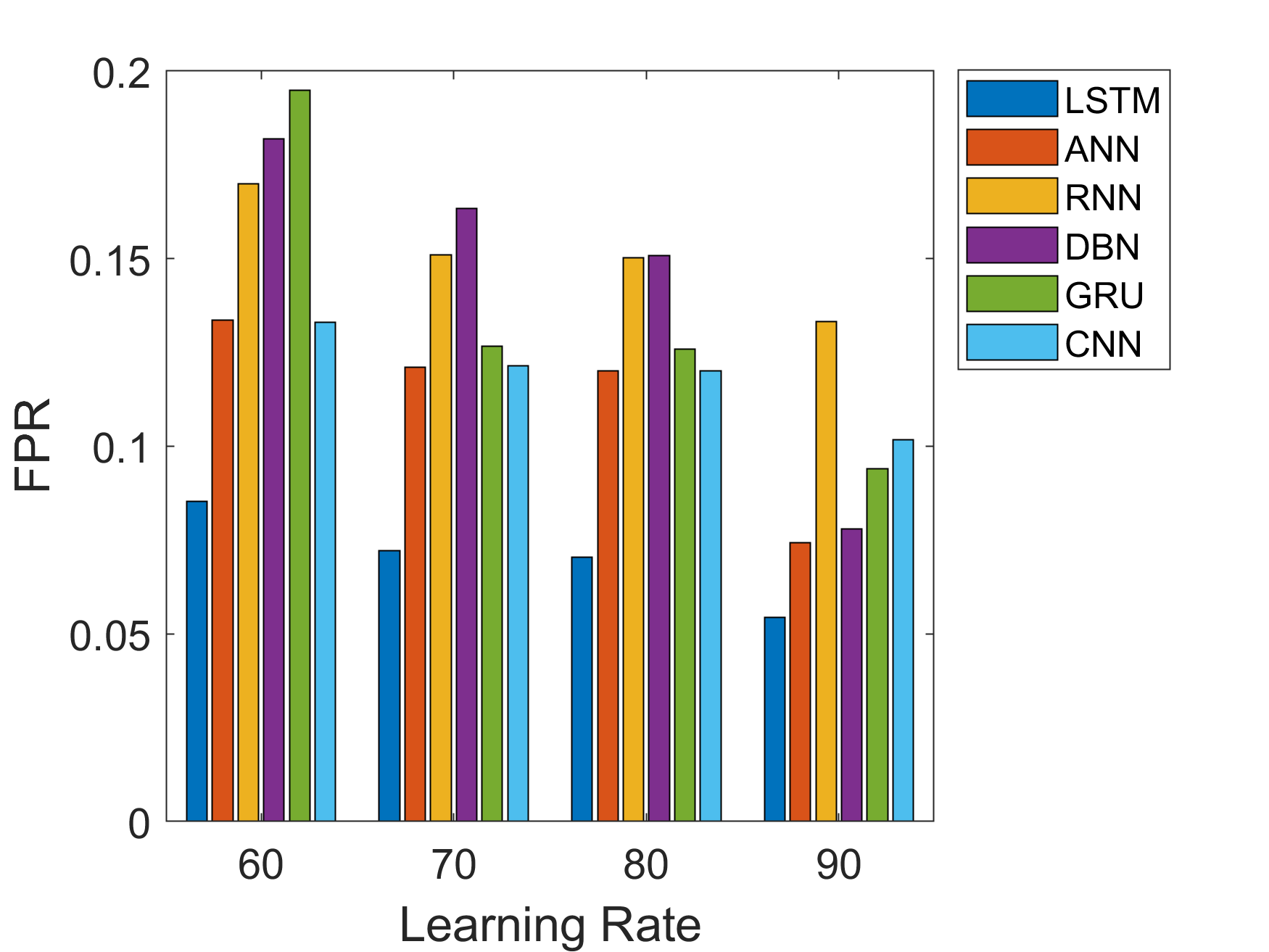

Supplement: S1 File — (ZIP) [file pone.0289306.s001.zip › Supporting Information files/FPR.tif]
